# Genetic regulation of disease risk and endometrial gene expression highlights potential target genes for endometriosis and polycystic ovarian syndrome

**DOI:** 10.1038/s41598-018-29462-y

**Published:** 2018-07-30

**Authors:** Jenny N. Fung, Sally Mortlock, Jane E. Girling, Sarah J. Holdsworth-Carson, Wan Tinn Teh, Zhihong Zhu, Samuel W. Lukowski, Brett D. McKinnon, Allan McRae, Jian Yang, Martin Healey, Joseph E. Powell, Peter A. W. Rogers, Grant W. Montgomery

**Affiliations:** 1 The Institute for Molecular Bioscience, The University of Queensland, Brisbane, QLD 4072 Australia; 2 Gynaecology Research Centre, The University of Melbourne, Department of Obstetrics and Gynaecology, Royal Women’s Hospital, Parkville, VIC 3052 Australia; 3 Department of Obstetrics and Gynaecology, University hospital of Berne, 3010 Berne, Switzerland; 4 Department of Anatomy, University of Otago, Dunedin, New Zealand

## Abstract

Gene expression varies markedly across the menstrual cycle and expression levels for many genes are under genetic control. We analyzed gene expression and mapped expression quantitative trait loci (eQTLs) in endometrial tissue samples from 229 women and then analyzed the overlap of endometrial eQTL signals with genomic regions associated with endometriosis and other reproductive traits. We observed a total of 45,923 *cis*-eQTLs for 417 unique genes and 2,968 *trans*-eQTLs affecting 82 unique genes. Two eQTLs were located in known risk regions for endometriosis including *LINC00339* on chromosome 1 and *VEZT* on chromosome 12 and there was evidence for eQTLs that may be target genes in genomic regions associated with other reproductive diseases. Dynamic changes in expression of individual genes across cycle include alterations in both mean expression and transcriptional silencing. Significant effects of cycle stage on mean expression levels were observed for (2,427/15,262) probes with detectable expression in at least 90% of samples and for (2,877/9,626) probes expressed in some, but not all samples. Pathway analysis supports similar biological control of both altered expression levels and transcriptional silencing. Taken together, these data identify strong genetic effects on genes with diverse functions in human endometrium and provide a platform for better understanding genetic effects on endometrial-related pathologies.

## Introduction

Variation in gene expression in human endometrium is strongly influenced by stage of the menstrual cycle^1,2^ and subject to the effects of genetic variation^3^. Understanding regulation of gene expression in this tissue is important because the endometrium is essential for female fertility including the establishment and maintenance of pregnancy^4,5^. Each menstrual cycle, under the influence of circulating steroid hormones, the endometrium regenerates with changes in cellular and molecular events in preparation for possible pregnancy^2,6,7^.

Common genetic effects alter expression of many genes and are known as expression quantitative traits (eQTLs). The eQTLs play an important role in mediating effects of genetic factors increasing risk for common diseases^8,9^. The genetic effects may be tissue specific or influence expression across multiple tissues, and may interact with other factors including changing hormonal environments^10,11^. Major international projects like The Genotype-Tissue Expression (GTEx) project^12,13^ and the Epigenetic RoadMap^14^ are designed to identify eQTLs and understand genetic regulation of gene expression across multiple tissues and cell types. Results from the latest GTEx study in more than 400 samples across 42 distinct tissues show local *cis*-acting genetic variants tend to be of two classes, either affecting most tissues or active in only a small number of tissues^12^. In contrast, *trans*-eQTL effects tend to be tissue-specific and enriched in enhancer regions^12^.

We analyzed genetic regulation of gene expression in endometrium, a tissue not included in the GTEx study, and the overlap of endometrial eQTL signals with signals for genetic risk factors in genomic regions associated with endometriosis and other reproductive traits available in GWAS catalogue including endometrial cancer and Polycystic ovary syndrome (PCOS). Endometriosis is a common disease affecting 7–10% of women^15^. The endometrium is considered an important source of cells that initiate the peritoneal lesions characteristic of endometriosis^16,17^ and we initiated studies of genetic regulation of gene expression in the endometrium as part of functional analyses to follow up genomic regions associated with endometriosis risk^18–23^. Our initial study identified eQTLs for 198 unique genes in endometrium^3^. The aims of this study were to expand the sample size to increase the power of our eQTL studies in endometrium and conduct formal analyses of the overlap between endometrial eQTLs and genetic variants associated with risk for endometriosis and other reproductive traits.

## Results

### Identification of complex structure of gene expression data in endometrium

**Figure 1:**
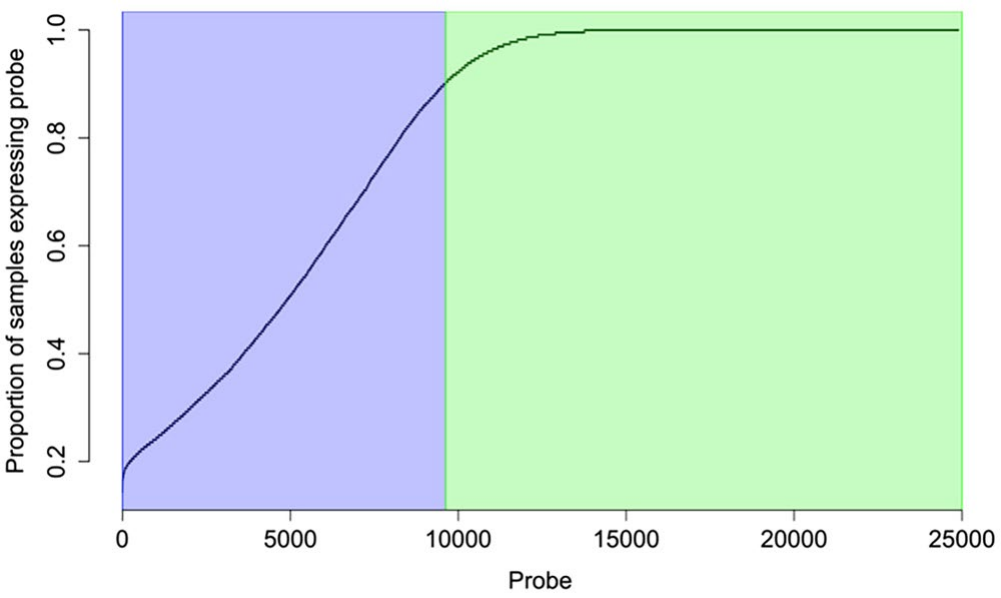
Variation in the proportion of samples expressing individual probes for probes expressed above background in one or more individuals. The region shaded in purple shows genes expressed in variable proportions of samples and the region shaded in green shows probes expressed in ≥90% of all samples.

We analysed gene expression in endometrial samples collected from 229 women of European ancestry attending clinics at the Royal Women’s Hospital in Melbourne (the RWH dataset; *n* = 165) and Melbourne IVF in Melbourne (the IVF dataset; *n* = 64). Principle component analysis (PCA) of overall gene expression showed both sample groups cluster together within the same stages of the menstrual cycle with no apparent differences in the overall expression levels of genes between the two sample groups (Fig. S1a,b). Most of the IVF samples were collected during the early and mid-secretory phases of the cycle. Samples at this stage of the cycle clustered well together with early and mid-secretory phase samples from the RWH set (Fig. S1c). Some IVF patients (29/64) had an IVF cycle prior to the sample collection cycle. We did not detect any significant differences in gene expression, or in the proportions of samples expressing different genes between samples with or without an IVF cycle treatment prior to biopsy. Therefore, RWH and IVF samples were combined for subsequent analyses. On average 43% of probes were expressed above background in individual samples with little variation between individuals (variance = 0.0003) (Fig. S2). However, we did observe substantial variability in the proportion of samples expressing individual probes (variance = 0.2005) (Fig. 1). 15,262 probes, mapping to 12,321 unique genes, were expressed in ≥90% of all samples. In contrast, 9,626 probes, mapping to 7,567 unique genes, with non-zero expression in at least one sample showed variation in the proportion of samples with non-zero expression; range 1–90% of samples (Fig. 1). Given the complex structure of gene expression in the endometrium, we conducted separate analyses for these two sets of probes in our subsequent studies (Fig. 1).

### Changes in gene expression across the menstrual cycle

Stage of the menstrual cycle was determined for each sample from histological assessment of sections of endometrial tissue by an experienced pathologist. Individuals were assigned to one of seven stages of the menstrual cycle based on histological classification as described in the methods. Inclusion and exclusion criteria are described in the methods.

**Figure 2:**
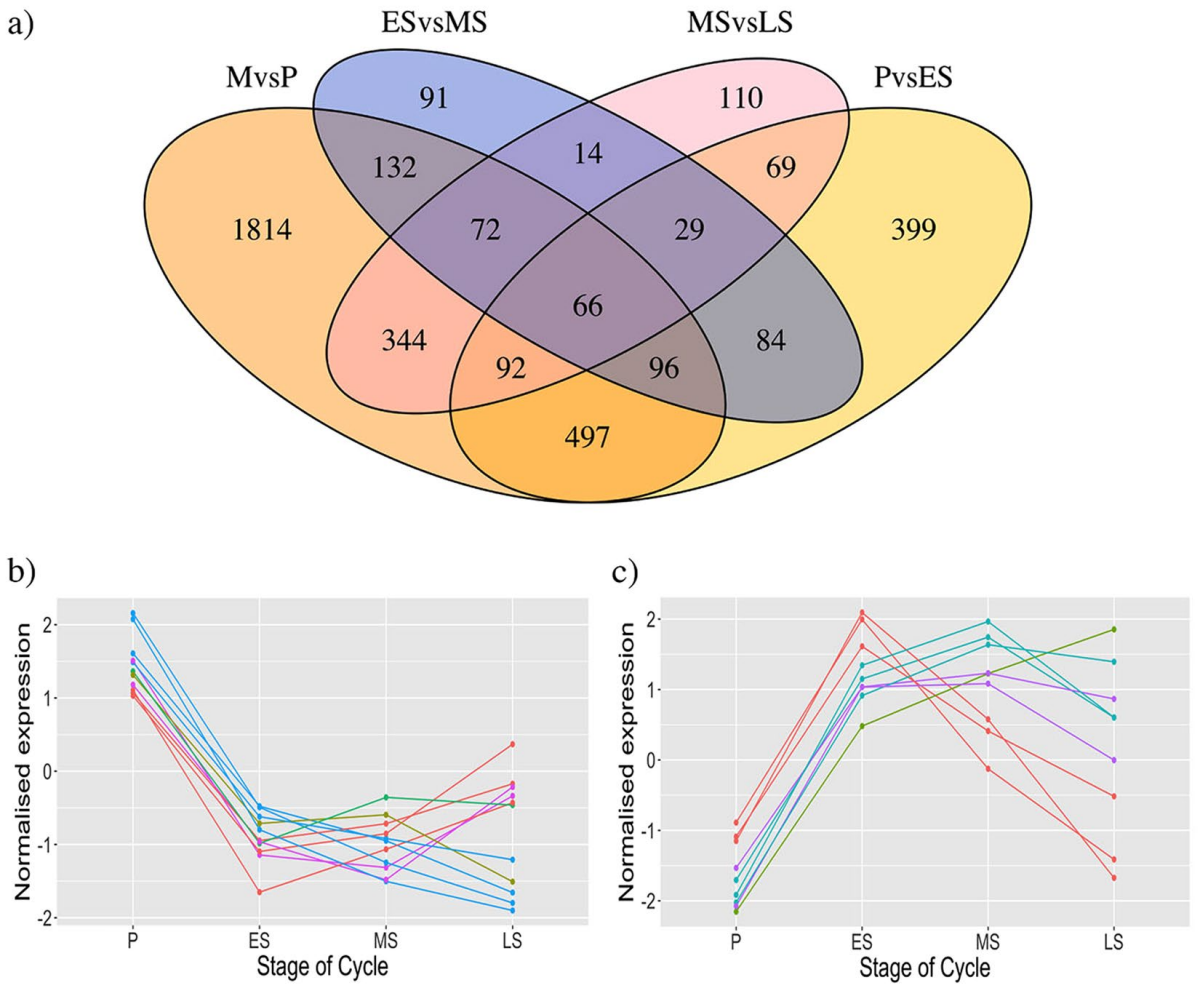
(**a**) The Venn diagrams showing the number of significant differentially expressed genes across the menstrual cycle between the menstrual (M) and proliferative (P) phases (orange), proliferative and early secretory (ES) phases (yellow), early and mid-secretory (MS) phases (blue), mid and late-secretory (LS) phases (pink) and overlapping probes between sets. (**b**,**c**) The line graphs showing gene expression patterns of the top differentially expressed genes across the cycle (P to LS phases).

We analyzed differences across the menstrual cycle in mean expression for the 12,321 genes (15,262 probes) expressed in ≥90% of samples and adjusted the false discovery rate (FDR) using the Benjamini-Hochberg multiple testing correction. Preliminary analyses identified few differences in gene expression between women in early proliferative (EP), mid proliferative (MP) and late proliferative (LP) stages of the cycle and these were combined as proliferative (P) stage for most subsequent analyses (Table S1). Details of all differentially expressed genes across the menstrual cycle are given in Tables S2–5. The number of significant differentially expressed genes across the menstrual cycle between M vs. P, P vs. ES, ES vs. MS and MS vs. LS and overlapping probes between sets are summarized in Fig. 2a. The majority of the differentially expressed genes were the same as those we reported previously^3^ (Fig. S3). Patterns of change for individual genes significantly down-regulated (Fig. 2b) or up-regulated (Fig. 2c) between P and ES demonstrate the dynamic and variable nature of changes for individual genes across the menstrual cycle.

**Figure 3:**
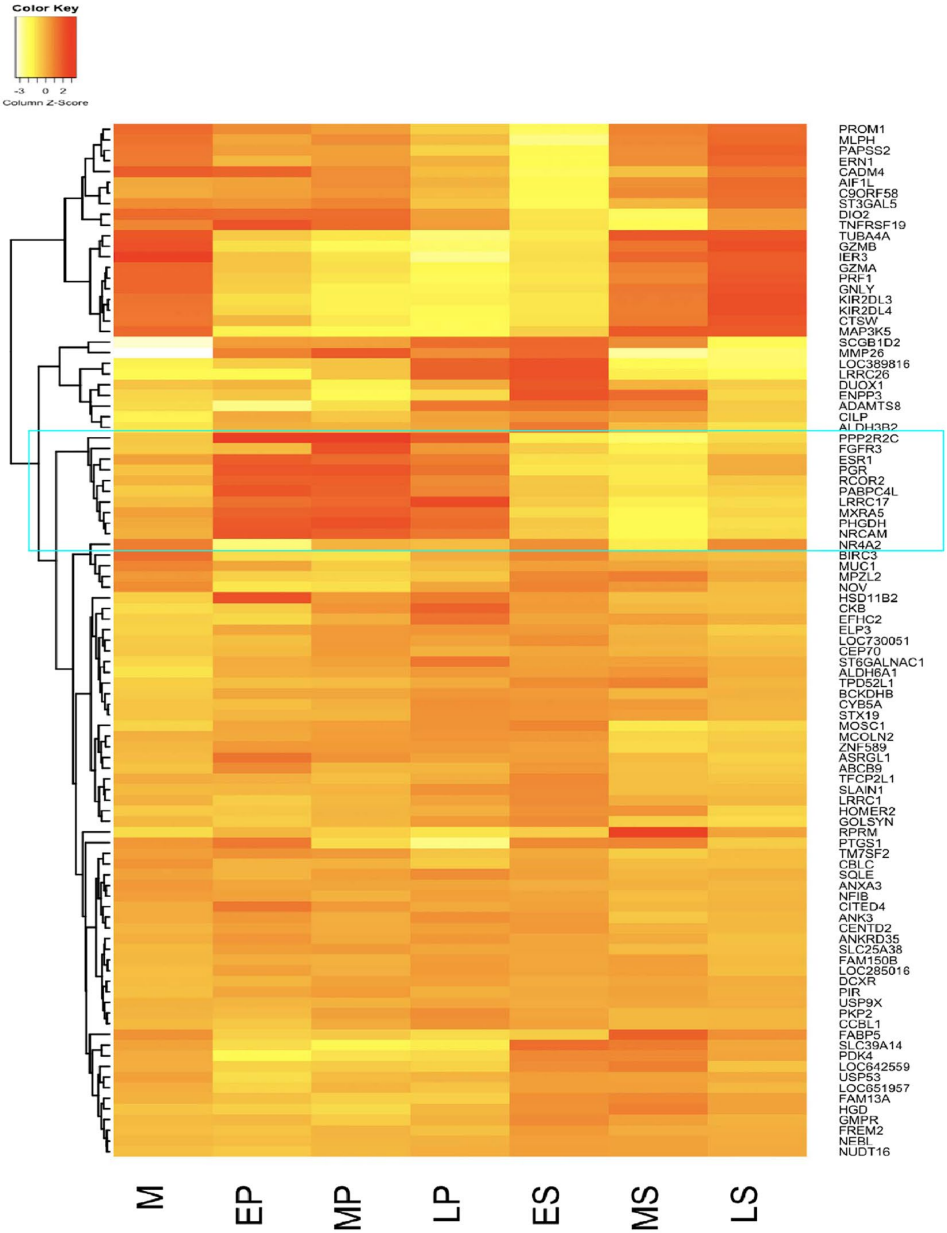
The heatmap showing the gene expression profile of 95 unique genes with marked changes across the menstrual cycle (Menstrual (M), Proliferative (P), Early- Secretory (ES), Mid-Secretory (MS) to Late-Secretory (LS) phases) including oestrogen receptor (*ESR*) and progesterone receptor (*PGR*). The genes clustered with *ESR* and *PGR* are highlighted in the blue box.

The most dynamic changes included 95 genes (109 probes) with significant differences in expression progressively across each stage of the menstrual cycle (Fig. 3). Estrogen receptor 1 (*ESR1*) and progesterone receptor (*PGR*) showed similar expression profiles across the menstrual cycle and by clustering expression of these 95 genes with *ESR1* and *PGR*, we observed sets of genes with differential patterns of expression. This included genes with similar or opposite expression patterns to *ESR1* and *PGR*, or patterns unrelated to expression of the receptors (Fig. 3). Nine genes, including *PPP2R2C*, *FGFR3*, *RCOR2*, *PABPC4L*, *LRRC17*, *MXA5*, *PHGDH*, *NRCAM* cluster with *ESR1* and *PGR* and show similar changes in gene expression across the menstrual cycle (Fig. 3).

### Variation in genes expressed/not-expressed in women at different menstrual cycle stages

Approximately 30% of the probes not expressed in all samples (2,877/9,626) show significant differences in the proportions of samples expressing these probes at different stages of the menstrual cycle. Preliminary analyses showed there were no significant differences in the proportion of expressed/not expressed genes between EP vs. MP, MP vs. LP, EP vs. LP or EP + MP vs. LP and these were combined as proliferative (P) stage for most subsequent analyses (Table S1). The largest number of probes showing altered expression was observed between the P and ES stages with expression of 1,186 probes activated between the ES and P stages of the cycle and expression of 1,323 probes repressed between the ES and P stages of the cycle (Table S6). Significant differences were also observed between M and P stages with expression of 218 probes repressed and 201 probes activated in M stage (Table S7) and ES and MS stages with expression of 214 probes repressed and 163 probes activated between the ES and MS stages of the cycle (Table S8). Only a small number of probes were shown to differ between MS and LS with expression of 34 probes repressed and 16 activated between the MS and LS stages (Table S9).

**Figure 4:**
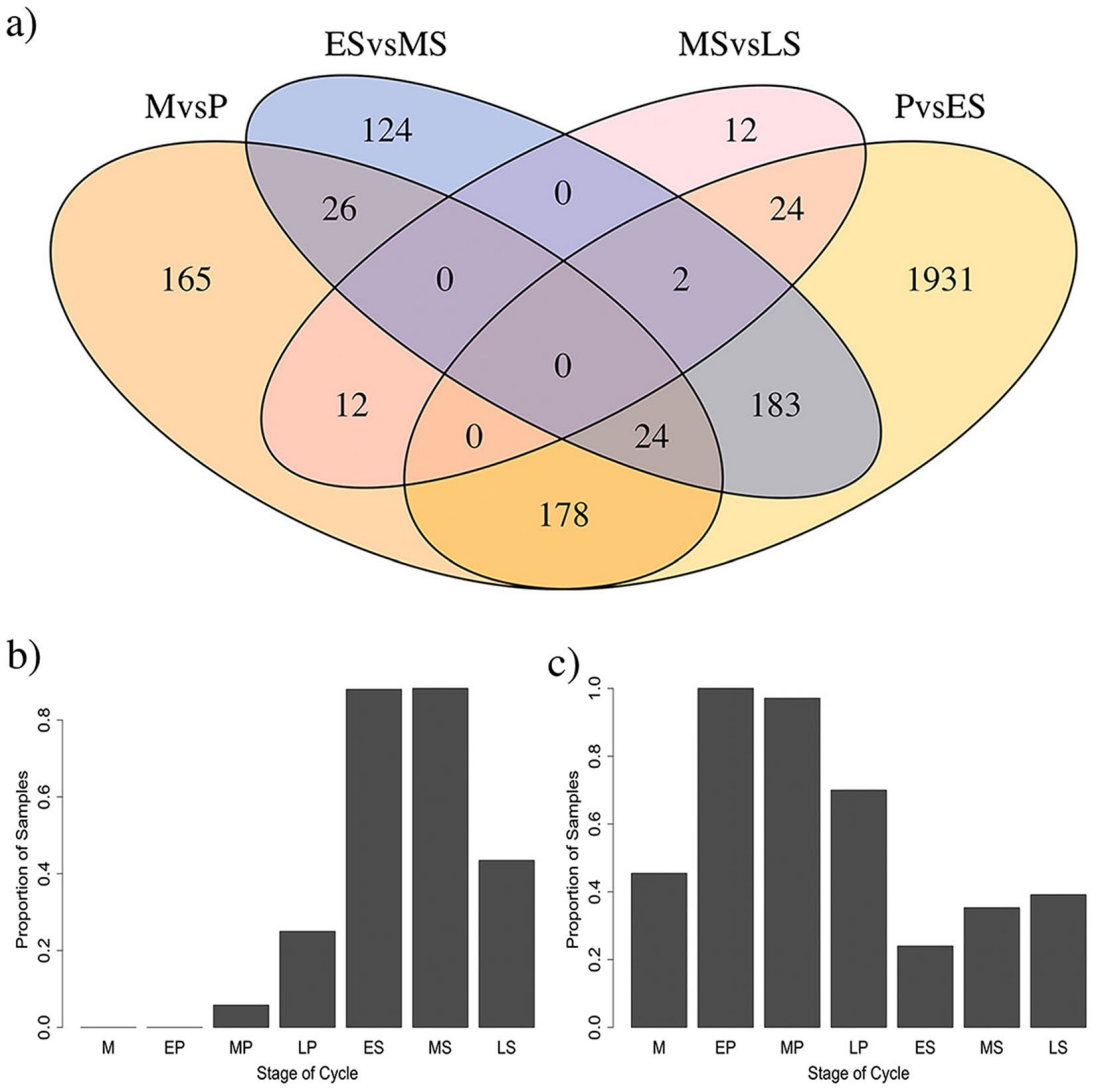
Probes expressed in different proportions of samples across the menstrual cycle. (**a**) The Venn diagrams showing the number of genes expressed in a significantly different proportion of samples across the menstrual cycle between the menstrual (M) and proliferative (P) phases (orange), proliferative and early secretory (ES) phases (yellow), early and mid-secretory (MS) phases (blue), mid and late-secretory (LS) phases (pink) and overlapping probes between sets. (**b**) Proportion of samples from each stage of the cycle expressing *ANGPTL1*. (**c**) Proportion of samples from each stage of the cycle expressing *OGDHL*.

Table 1 shows the most significant probes activated or repressed between stages. There was overlap between genes/probes expressed in different proportions of individuals between the P and ES stages and between the ES vs. MS and MS vs. LS stages of the cycle (Fig. 4a). Two genes showing profound differences in proportions of samples expressing these genes across the cycle were *ANGPTL1* and *OGDHL* (Fig. 4b,c). *ANGPTL1* was expressed in over 80% of ES samples and very few proliferative samples, whilst *OGDHL* was expressed in close to 100% of early proliferative stage samples and <30% of ES samples.

**Table 1:** Top 30 probes showing significant differences in the proportion of samples in which they are expressed across the menstrual cycle.

Probe	Gene	P-value	Adjusted P-value	Cycle stage effect
ILMN_1669773	*ANGPTL1*	1.48E-13	1.40E-09	repressed P and activated ES
ILMN_1683923	*MT1H*	5.53E-13	1.40E-09	repressed P and activated ES
ILMN_1688580	*CAMP*	5.91E-13	1.40E-09	repressed P and activated ES
ILMN_2099315	*TRPM8*	6.01E-13	1.40E-09	repressed P and activated ES
ILMN_2056815	*LINGO4*	7.35E-13	1.40E-09	repressed P and activated ES
ILMN_1714577	*OGDHL*	4.60E-12	2.46E-09	activated P and repressed ES
ILMN_1779685	*ACCN1*	5.91E-12	2.96E-09	activated P and repressed ES
ILMN_2077952	*GALNTL1*	9.05E-12	3.96E-09	activated P and repressed ES
ILMN_2185675	*FAM159A*	1.47E-11	5.78E-09	activated P and repressed ES
ILMN_1656192	*ZNF704*	2.55E-11	8.46E-09	activated P and repressed ES
ILMN_2289593	*FXYD2*	7.93E-09	4.27E-05	repressed ES and activated MS
ILMN_2385416	*GPX5*	9.90E-09	4.27E-05	repressed ES and activated MS
ILMN_1728327	*LOC150577*	1.92E-07	0.000299781	repressed ES and activated MS
ILMN_1787932	*GPR110*	2.18E-07	0.000299781	repressed ES and activated MS
ILMN_1787266	*SPINK1*	2.10E-07	0.000299781	repressed ES and activated MS
ILMN_1734472	*PEBP4*	1.87E-07	0.000299781	activated ES and repressed MS
ILMN_1792404	*TM4SF4*	4.64E-07	0.000402866	activated ES and repressed MS
ILMN_1685496	*RGS7*	8.43E-07	0.000402866	activated ES and repressed MS
ILMN_1789040	*SLITRK5*	8.65E-07	0.000402866	activated ES and repressed MS
ILMN_1799335	*PCDHA6*	9.75E-07	0.000402866	activated ES and repressed MS
ILMN_1669123	*C1ORF187*	2.33E-06	0.01121429	repressed MS and activated LS
ILMN_1708348	*ADAM8*	3.16E-05	0.030642767	repressed MS and activated LS
ILMN_2298159	*PRDM1*	3.53E-05	0.030642767	repressed MS and activated LS
ILMN_1765994	*ZBP1*	5.73E-05	0.030642767	repressed MS and activated LS
ILMN_1788817	*MAGED4B*	6.76E-05	0.034248295	repressed MS and activated LS
ILMN_1660729	*ATP6V1C2*	1.87E-06	0.01121429	activated MS repressed LS
ILMN_1717886	*PKHD1L1*	2.07E-05	0.030642767	activated MS repressed LS
ILMN_2067596	*KCNS2*	2.11E-05	0.030642767	activated MS repressed LS
ILMN_2090641	*FAM110C*	2.33E-05	0.030642767	activated MS repressed LS
ILMN_1663399	*TIMP4*	2.68E-05	0.030642767	activated MS repressed LS

The genes significantly up- or down-regulated across the menstrual cycle were analyzed in the GEN2FUNC module of the Functional Mapping and Annotation of Genome-Wide Association (FUMA) software (see methods). From FUMA, the significant hallmark pathways^24^ for genes with variable level of expression (adjusted p-value < 10^−12^) included ‘epithelial to mesenchymal transition (EMT)’, ‘oestrogen response late’, ‘oestrogen response early’, and ‘kras signalling up’ (Table 2). Hallmark pathways enriched for genes expressed in different proportions of samples across the cycle (adjusted p-value < 10^−10^) were very similar and included ‘oestrogen response early’, ‘oestrogen response late’, ‘e2f targets’, ‘kras signalling up’, and ‘epithelial to mesenchymal transition’ (Table 2).

**Table 2:** Hallmark pathways enriched for DE genes and genes expressed in different proportions of samples across the P to LS phases.

	GeneSet	N	n	P-value	adjusted P
DE Genes	hallmark epithelial mesenchymal transition	199	89	1.21E-62	5.94E-61
	hallmark estrogen response late	200	73	3.52E-44	8.63E-43
	hallmark estrogen response early	200	68	5.63E-39	9.20E-38
	hallmark kras signaling up	199	67	4.01E-38	4.91E-37
	hallmark il2 stat5 signaling	200	62	4.69E-33	4.59E-32
	hallmark hypoxia	200	61	4.19E-32	3.42E-31
	hallmark apoptosis	161	50	3.81E-27	2.67E-26
	hallmark xenobiotic metabolism	200	54	9.73E-26	5.30E-25
	hallmark e2f targets	200	54	9.73E-26	5.30E-25
	hallmark glycolysis	200	53	7.15E-25	3.50E-24
Expressed/not expressed genes	hallmark estrogen response early	200	39	2.33E-14	5.70E-13
	hallmark estrogen response late	200	39	2.33E-14	5.70E-13
	hallmark e2f targets	200	38	1.15E-13	1.89E-12
	hallmark kras signaling up	199	36	2.21E-12	2.71E-11
	hallmark epithelial mesenchymal transition	199	35	1.00E-11	8.17E-11
	hallmark apical junction	199	35	1.00E-11	8.17E-11
	hallmark myogenesis	200	32	8.68E-10	6.08E-09
	hallmark xenobiotic metabolism	200	29	4.69E-08	2.56E-07
	hallmark g2m checkpoint	200	29	4.69E-08	2.56E-07
	hallmark kras signaling dn	200	27	5.55E-07	2.72E-06

N is the total number of genes in the pathway and n is the number of DE or expressed/not expressed genes in the pathway.

### Identification of endometrial *cis*-eQTLs and *trans*-eQTLs

**Figure 5:**
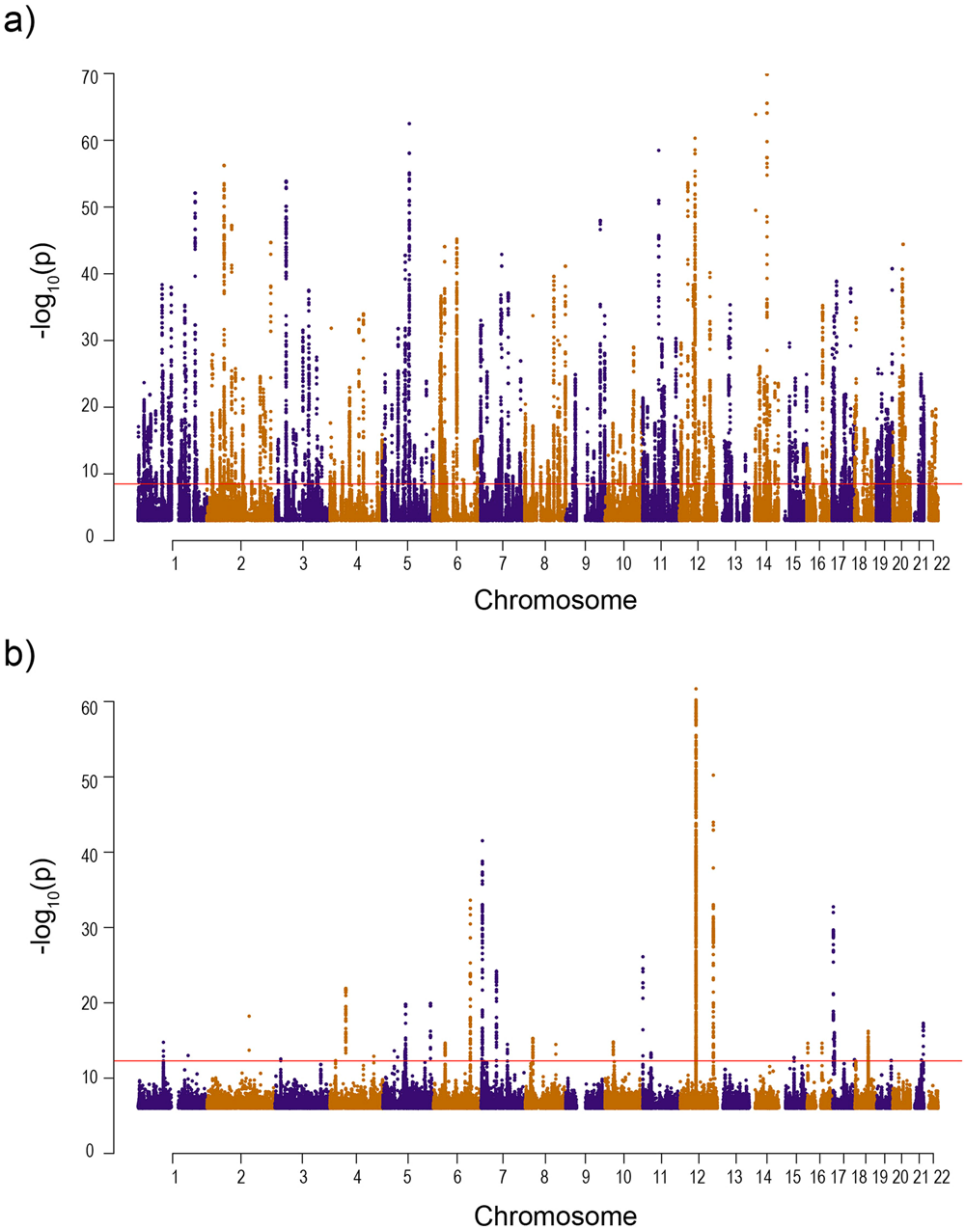
Manhattan plots of endometrial (**a**) *cis* and (**b**) *trans*-eQTLs. Each point represents an eSNP, chromosomes are defined by alternating purple and orange colours and the red line indicates a Bonferroni threshold of *p* < 3.3 × 10^−9^ for *cis*-eQTLs and *p* < 5.5 × 10^−13^ for *trans*-eQTLs.

We ran the eQTL analysis on the newly recruited samples of this study and compared to the eQTL results from our previous study^3^. Our results showed that all the eQTLs with p < 1 × 10^−3^ replicated with the same direction of effect between the new sample group and the samples analysed in our previous eQTL study^3^. In the combined analysis, we identified a total of 222,854 *cis*-eQTLs for 3,089 probes, which map to 2,758 unique genes at a FDR of 0.05 (Table 3). When a more stringent Bonferroni genome-wide significance threshold of *p* < 3.3 × 10^−9^ was applied, the number of significant *cis*-eQTLs reduces to 45,923 *cis*-eQTLs across 453 probes (417 unique genes) (Fig. 5a, Table S10). The 30 most significant *cis*-eQTLs are presented in Table 4. These results are publically available to browse or download at http://reproductivegenomics.com.au/shiny/eeqtl2/. Conditional analysis on 3,089 sentinel *cis*-eQTLs identified 336 secondary signals totalling 3,425 independent signals that mapped to 2,758 unique genes (Table S11). *Cis*-eQTLs were concentrated in positions close to transcription start sites (Fig. S4).

**Table 3:** Total number of *cis* and *trans*-eQTLs detected in endometrium using either FDR correction of 0.05 or Bonferroni correction.

eQTLs	No. pass FDR 0.05			No. pass Bonferroni		
	eQTLs	Unique probes	Unique genes	eQTLs	Unique probes	Unique genes
Total cis-eQTLs	222,854	3,089	2,758	45,923	453	417
Independent cis-eQTLs	3,425	3,089	2,758	469	453	417
Total trans-eQTLs	8771	854	774	2,968	89	82
Sentinel trans-eQTLs	1,593	854	774	104	89	82

**Table 4:** Top 30 most significant *cis* eQTLs in endometrium.

Probe ID	Gene Name	CHR	SNP	BP	A1	BETA	SE	P	Distance (bp)
ILMN_1798177	*CHURC1*	14	rs10142379	65354946	G	1.269	0.04766	1.35E-70	−46550
ILMN_3271179	*RP11-82H13.2*	14	rs2766	24686145	T	1.873	0.07653	1.32E-64	26
ILMN_1743145	*ERAP2*	5	rs2927608	96252432	A	1.831	0.07502	3.21E-63	3368
ILMN_1695585	*RPS26*	12	rs1131017	56435929	C	0.6456	0.02778	4.86E-61	−1280
ILMN_1765332	*TIMM10*	11	rs2848626	57283988	C	−0.8337	0.03686	3.29E-59	−12029
ILMN_1754501	*KIAA1841*	2	rs3213944	61372298	G	0.9616	0.044	6.04E-57	−17967
ILMN_2404850	*RPL14*	3	rs2276870	40499182	C	0.7358	0.03492	1.38E-54	−4596
ILMN_1753164	*IPO8*	12	rs10843810	30819597	C	0.6937	0.03305	2.37E-54	37338
ILMN_3299955	*RPS26*	12	rs1131017	56435929	C	0.4524	0.02161	3.51E-54	−406
ILMN_2352023	*DSTYK*	1	rs113817010	205104581	A	−0.816	0.03981	7.68E-53	−7169
ILMN_3285153	*RPS26*	12	rs1131017	56435929	C	0.7216	0.03572	6.18E-52	−1240
ILMN_3236498	*PSMD5-AS1*	9	rs10818476	123572038	A	1.044	0.05423	1.06E-48	−44172
ILMN_2173294	*THNSL2*	2	rs6547752	88447437	G	−1.054	0.05563	5.95E-48	−38618
ILMN_3242288	*RPS26*	12	rs1131017	56435929	C	0.6965	0.03717	2.19E-47	−1252
ILMN_2200659	*SNHG5*	6	rs1059307	86387888	G	−0.6661	0.03645	6.31E-46	−524
ILMN_2198408	*MFF*	2	rs58670479	228192473	T	1.255	0.06907	2.09E-45	−29792
ILMN_3235326	*SNHG17*	20	rs1739651	37048135	A	1.022	0.05669	3.91E-45	−1360
ILMN_2209027	*RPS26*	12	rs1131017	56435929	C	0.6352	0.03535	5.60E-45	−298
ILMN_1683279	*PEX6*	6	rs9471975	42919222	T	−0.8114	0.0453	8.84E-45	−12678
ILMN_1805377	*POMZP3*	7	rs6979487	76131645	A	1.254	0.0715	1.28E-43	−107658
ILMN_1772459	*RPS23*	5	rs73138787	81568934	A	−0.8894	0.05083	1.80E-43	−355
ILMN_2370872	*GRINA*	8	rs56261297	145066853	T	−0.3998	0.02353	7.39E-42	−582
ILMN_3209193	*RPS26*	12	rs11171739	56470625	C	0.5832	0.03437	8.71E-42	34410
ILMN_3268403	*ZNF667-AS1*	19	rs35215648	56983716	C	0.7641	0.04529	1.78E-41	−21847
ILMN_1670841	*CPNE1*	20	rs200929686	34198350	CA	−0.9118	0.05413	2.17E-41	−15771
ILMN_1719064	*KCTD10*	12	rs4766601	109890080	C	0.6889	0.04129	6.97E-41	3455
ILMN_2327994	*AZIN1*	8	rs1991927	103858748	T	−0.8619	0.05221	2.57E-40	19891
ILMN_3298167	*ZSWIM7*	17	rs6416868	15924370	A	−0.5671	0.03482	1.30E-39	44425
ILMN_1653794	*SNHG5*	6	rs3087978	86388223	C	−0.515	0.03173	2.00E-39	1042
ILMN_2325028	*ODF2L*	1	rs272489	86807618	C	0.885	0.0536	4.42E-39	−12605

**Figure 6:**
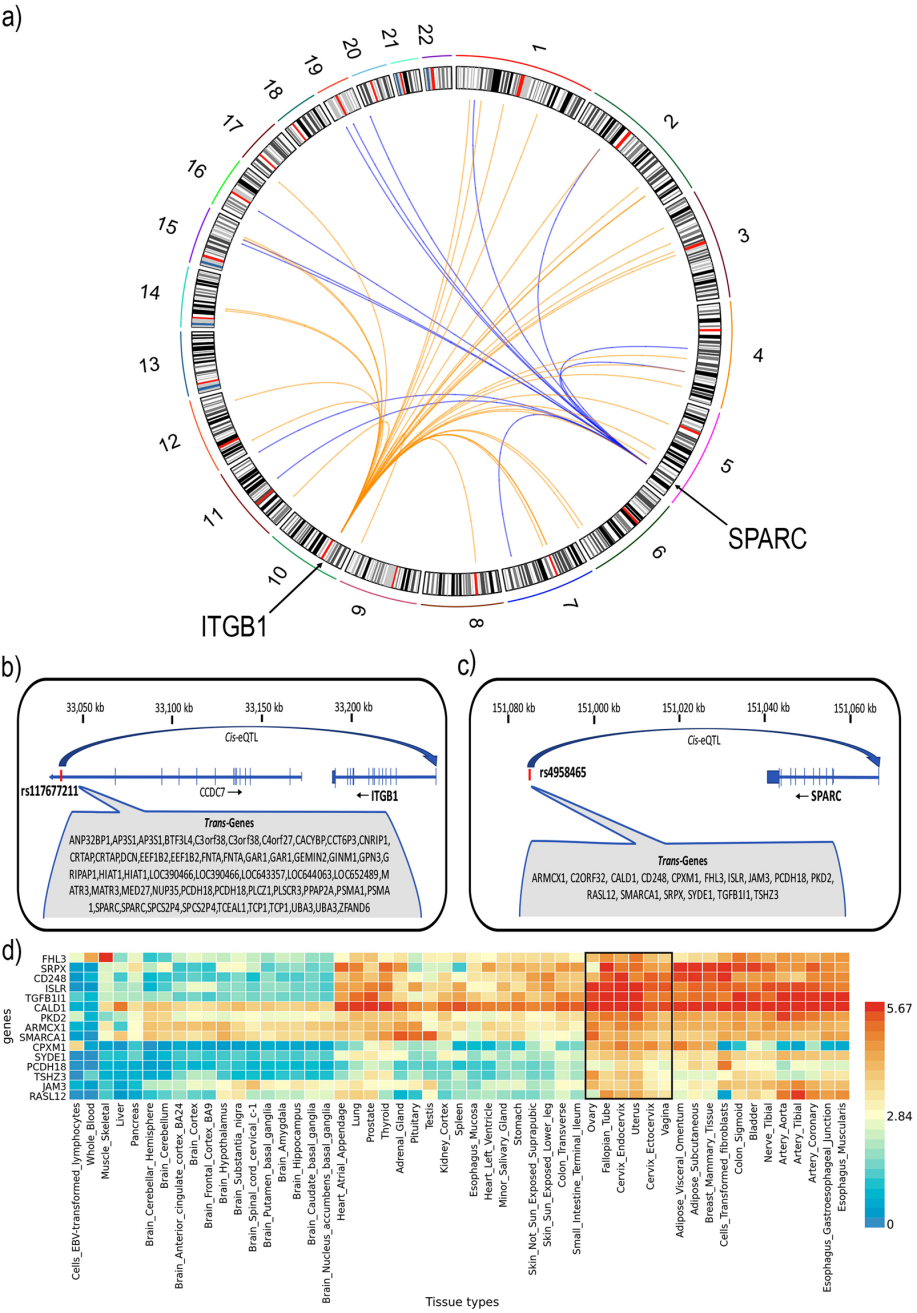
(**a**) Circos plot of the overlapping *cis* and *trans*-eQTLs on chromosome 5 (rs4958465) and 10 (rs117677211). Blue lines in the centre connect rs4958465 to genes with effects in *trans* and orange lines connect rs117677211 to genes that it effects in *trans*. (**b**) rs117677211-*ITGB1 cis*-eQTL on chromosome 10 and the genes that it effects in *trans*. (**c**) rs4958465-*SPARC cis*-eQTL on chromosome 5 and the genes that it effects in *trans*. (**d**) Heatmap of tissue specific expression of rs4958465 *cis* and *trans* genes, female reproductive tissues outlined in black.

We identified 8,771 *trans*-eQTLs using the FDR significance threshold of 0.05, including 1,593 sentinel signals across 854 probes (774 unique genes) (Table 3). The 30 most significant *trans*-eQTLs are presented in Table 5. Following Bonferroni genome-wide correction (*p* < 5.4 × 10^−13^), 2,968 *trans*-eQTLs remained affecting 89 probes and 82 unique genes (Fig. 5b, Table S12). We looked to see if *trans* eSNPs (eSNPs - SNP with a significant eQTL) also influenced expression of genes in the immediate region (were also *cis*-eSNPs). We observed overlap between 36 *trans*-eSNPs and *cis*-eSNPs in the endometrium, two of which affect genes that have been associated to endometrial biology^25–31^ and are shown in Fig. 6a. The location of the *ITGB1* and *SPARC cis*-eQTL and the *trans*-genes associated with the eSNP are shown in Fig. 6b,c respectively. Expression of genes associated with eSNP rs4958465 for *SPARC* was investigated across 53 tissues using FUMA software, expression patterns were found to be similar across female reproductive tissues (Fig. 6d). The overlapping *cis* and *trans*-eQTL affecting the largest number of genes was located on chromosome 18 in a region enriched for H3K4me1 histone marks and affected 269 unique probes (Fig. S5).

**Table 5:** Top 30 most significant *trans* eQTLs in endometrium.

SNP CHR	SNP	BP	Probe CHR	Probe ID	Gene Name	BETA	SE	P
12	rs1131017	56435929	19	ILMN_3254492	*RPS26P55*	0.84	0.03543	2.09E-62
12	rs1131017	56435929	1	ILMN_1726647	*RPS26P15*	0.6449	0.02779	6.21E-61
12	rs1131017	56435929	1	ILMN_3248833	*RPS26P15*	0.7986	0.03444	6.92E-61
12	rs1131017	56435929	9	ILMN_3290019	*RPS26P2*	0.9057	0.03919	1.17E-60
12	rs1131017	56435929	X	ILMN_2180866	*RPS26P11*	0.6742	0.02925	1.79E-60
12	rs1131017	56435929	18	ILMN_1737991	*RPS26P54*	0.9733	0.04241	3.41E-60
12	rs1131017	56435929	17	ILMN_3296994	*RP11-713H12.2*	0.8441	0.03695	7.03E-60
12	rs1131017	56435929	7	ILMN_1750636	*RPS26P47*	0.7471	0.03279	1.05E-59
12	rs1131017	56435929	13	ILMN_2310703	*RPS26P47*	0.7499	0.03301	1.69E-59
12	rs1131017	56435929	1	ILMN_3236675	*RPS26P13*	0.8887	0.04185	4.42E-55
12	rs1131017	56435929	8	ILMN_1677697	*RPS26P35*	0.7236	0.03543	1.30E-52
12	rs1131017	56435929	8	ILMN_1657950	*RP11-777J24.1*	1.01	0.04977	3.79E-52
12	rs1051470	118583232	2	ILMN_3285785	*PEBP1P2*	−1.398	0.07031	6.02E-51
12	rs1131017	56435929	10	ILMN_3190596	*RP11-57C13.5*	0.8352	0.04231	1.66E-50
12	rs11171739	56470625	15	ILMN_1678522	*RP11-330L19.1*	0.6171	0.03342	1.60E-46
12	rs1131017	56435929	18	ILMN_3291511	*RPS26P54*	0.7236	0.04157	3.51E-43
7	rs7612	5567112	5	ILMN_3235221	*CTC-512J14.7*	1.422	0.0831	3.05E-42
12	rs1131017	56435929	11	ILMN_3308808	*MIR130A*	0.7658	0.05164	1.21E-34
6	rs9483504	133135886	2	ILMN_1679920	*LOC651894*	0.5592	0.03816	2.39E-34
17	rs222757	3569913	9	ILMN_3260017	*HNRNPK*	−0.7662	0.05317	1.80E-33
11	rs866411223	810009	1	ILMN_1723433	*FAM72B*	−0.9104	0.07306	7.66E-27
7	rs563273497	56107789	1	ILMN_1704291	*CHCHD2P6*	−0.3612	0.03083	6.12E-25
4	rs35057235	57261024	3	ILMN_3236680	*PPATP1*	−0.3173	0.02889	1.22E-22
5	rs62381648	170806428	12	ILMN_1678775	*NPM1*	−0.1932	0.0187	1.24E-20
5	rs73138787	81568934	1	ILMN_1653039	*ANKRD65*	−0.3511	0.03412	1.62E-20
12	rs11171739	56470625	3	ILMN_3262348	*IP6K2*	0.4107	0.04032	3.44E-20
2	rs116743765	150935585	14	ILMN_1715607	*CHMP4A*	−0.6367	0.06515	5.95E-19
21	rs4819003	46405793	18	ILMN_3225894	*RP11-757O6.4*	−0.4413	0.04667	5.14E-18
18	rs79045919	47697194	14	ILMN_1758543	*CNIH*	−1.312	0.1444	6.07E-17
17	rs222851	7139238	3	ILMN_3298824	*LOC728787*	0.3601	0.0398	8.95E-17

### Functional annotations of gene sets

#### Oestrogen receptor (ESR1) binding

We tested for overlap between eSNPs and ESR binding sites and identified 26 *cis* eSNPs and one *trans* eSNPs that were within known ESR binding sites (Table S13). Approximately 43% (905/2095) of genes differentially expressed across the menstrual cycle and 38% (945/2516) of genes expressed in different proportions of samples across the cycle contained ESR binding sites within 50 kb of their transcription start site (Tables S14 and 15). A separate pathway analysis conducted on genes with ESR binding sites within 50 kb of their TSS showed a more significant enrichment of the ‘early and late oestrogen response’ pathways (Fig. S6).

#### Transcription factors

We demonstrated that 3366 *cis*-eSNPs regulate expression of 41 transcription factors and 2 *trans*-eSNPs regulate expression of 2 transcription factors including *RBM7* and *BTF3* (Table S16). *Cis*-eQTLs that regulate transcription factors may generate associations in *trans* to transcription factor target genes. The SNP rs4970988 at chromosome 1 displayed a strong *cis*-association with *ARNT*. This SNP also showed a *trans*-association (*p* < 1 × 10^−5^) with genes at chromosomes 5,7,9,11,13 and 19 including *ZNF615, RNF20, WDR36, SAP18, ZNF467, ANKMY2, TMEM16A* and *GIN1*, although these *trans*-associations did not reach the study-wide significance for *trans*-eQTLs. About 10% of the significant differentially expressed genes across the menstrual cycle (208/2095) are transcription factors (Table S17) and about 8% (202/2516) of the significant expressed/not expressed genes across the menstrual cycle are transcription factors (Table S18).

### Alleles associated with genes expressed at different frequencies

Logistic regression was used to test for any association between genotype and whether a gene is expressed or not-expressed in different samples (eBTL analysis mentioned in methods). We detected 63 significant *cis* associations using an FDR cut-off of 0.05 (Table S19) and eight significant *cis* associations when using a more stringent Bonferroni genome-wide correction (p < 5.2 × 10^−9^) (Table 5, Fig. S7a). The effect of genotype on the proportion of samples expressing *MAG* and *VAPA* is shown in Fig. S7b,c, the G allele at both rs10411704 and rs627262 is associated with *MAG* and *VAPA* being expressed in samples, respectively. Examination of the probe positions relative to the transcripts showed that, in the case of *VAPA*, ILMN_2405190 binds to only one of several transcripts. A second probe for this gene (ILMN_1690822) targets an alternative transcript and has an eQTL (rs542215, *p* = *7.16* × 10^*−7*^); the G allele at both SNPs is associated with expression in an increased proportion of samples. Probes for *BRWD*2, *RPS6KA2* and *SEMA4G* showed genetic effects on transcriptional silencing, targeting extra gene transcripts when compared to the alternative probes targeting the same genes. These results suggest that some of the effects of genotypes on gene regulation are transcript specific (Table 6).

**Table 6:** Significant associations between genotypes and the proportion of samples expressing a probe in endometrium.

CHR	SNP	BP	A1	OR	SE	P-value	Probe ID	ILMN Gene ID
18	rs627262	9959370	A	0.1497	0.2942	1.07E-10	ILMN_2405190	*VAPA*
10	rs1659597	122610646	C	0.00766	0.775	3.25E-10	ILMN_2086222	*BRWD2*
16	rs382745	89603586	G	4.753	0.2484	3.49E-10	ILMN_1675583	*SPG7*
6	rs9347162	167271716	T	9.667	0.3621	3.73E-10	ILMN_1716218	*RPS6KA2*
19	rs10411704	35800662	G	10.6	0.3773	3.91E-10	ILMN_1803773	*MAG*
10	rs3740484	102747363	T	8.074	0.3374	5.98E-10	ILMN_1678974	*SEMA4G*
8	rs2906331	194884	T	0.1696	0.2942	1.63E-09	ILMN_2326376	*ZNF596*
9	rs568886	2532598	A	6.922	0.3279	3.65E-09	ILMN_3243324	*FLJ35024*

### Overlap between genes differentially expressed across the menstrual cycle and eQTLs

An important question is how genetic effects interact with physiological influences on gene expression. To address this, we looked at the distribution of eQTL genes across genes whose expression does or does not change across the cycle. *Cis*-eQTLs for 896 unique genes were also detected as differentially expressed across stages of the menstrual cycle (Fig. S8). The 36% overlap observed did not differ from the proportion expected by chance (chi-square statistic = 0.95, *p* = *0.33*). We next tested for context specific interactions between genotype and stage of cycle using 129 probes that met the more stringent Bonferroni genome-wide eQTL significance threshold and were differentially expressed across the menstrual cycle. For the 129 probes tested, no significant interactions were detected. This was however limited by sample number with alleles having relatively low minor allele frequencies not represented within all the stage groups tested for many probes. Post-hoc analysis with our limited clinical data did not identify other conditions influencing gene expression that may have biased our results.

### Endometrial eQTLs overlap blood eQTLs

**Figure 7:**
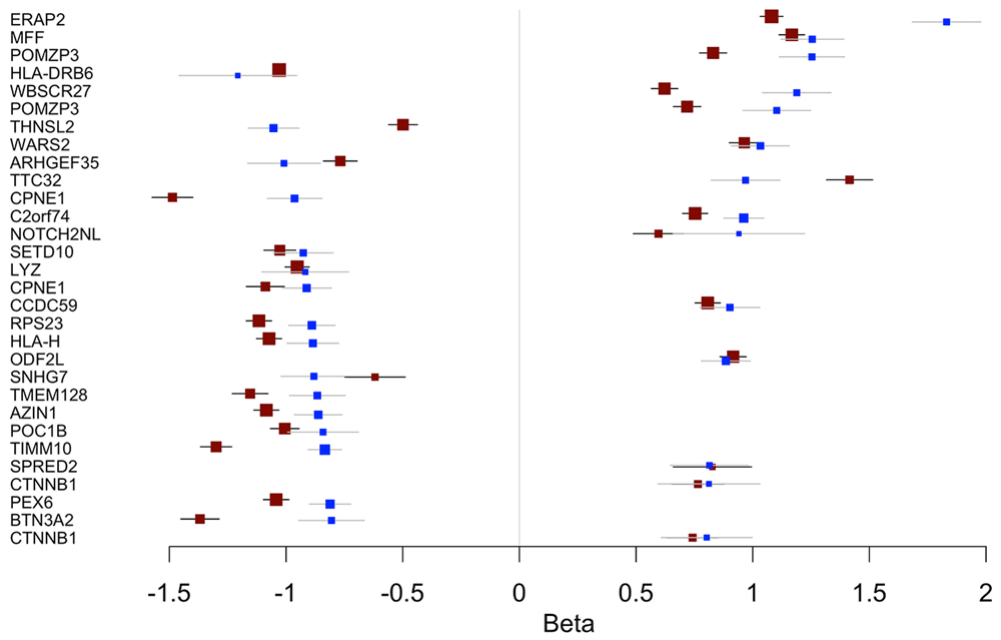
Effect size of the top 30 endometrial eQTLs of largest effect (blue) compared with effect sizes published in blood (red).

We looked to see if eQTLs reported in endometrium had also been reported in studies with whole blood. We identified 318 endometrial eQTLs overlapping blood eQTLs of which 294 had the same eSNP and the remaining probes were associated with SNPs in strong linkage equilibrium (r^2^ > 0.7) with the eSNP. Of these overlapping eQTLs, 301 were present in the CAGE dataset and an additional 17 not present in the CAGE data were recorded in the GTEx database. Overall ~68% of endometrial *cis*-eQTLs overlap with those identified in blood. eQTLs with the largest effect size in endometrium were shown to have the same directional effect in blood (Fig. 7).

### Endometriosis case/control analysis

We analysed endometriosis cases and controls for differences in the mean expression of genes/probes expressed in 90% of samples and for genes/probes expressed in variable numbers of samples in separate analyses. Some genes showed nominally significant differences in expression (Tables S20 and S21) after correction for effects of stage of the menstrual cycle. However, after correcting for multiple testing in each model, there were no genes with significantly different gene expression between endometriosis cases and controls for either analysis. This result did not change after correction for multiple testing with either a Bonferroni adjustment or Benjamini-Hochberg FDR < 0.05. To explore this result further, we looked at two genes where expression is reported to differ between endometriosis cases and controls^32,33^. Probes for both *HOXA*1*0* and *EMX2* were expressed in >90% of samples. Mean expression levels for these genes in endometriosis cases and controls were not significantly different (Figs S9a and S10a). Both genes show strong evidence of variation in expression across the cycle with higher expression in the proliferative compared with the secretory phase (*p* < 10^−12^; Figs S9b and S10b). We conducted analysis of the interaction between stage of the cycle and case control status. There was nominal significance for an interaction for *HOXA*10 (*p* = *0.04*) with expression of *HOXA10* remaining higher and more variable in cases in the late secretory phase of the cycle compared with controls (Fig. S9c,d). There was no evidence for an interaction between stage of the cycle and control status for *EMX2* (Fig. S10c,d).

### Overlap between eQTLs and GWAS signals

#### GWAS catalogue traits

We next sought to identify the degree of overlap between endometrial tissue eQTLs and GWAS loci, based upon a minimum linkage disequilibrium (LD) *r*^*2*^ > 0.7 between the eSNP and GWAS SNP in the 1000 Genome reference panel. Of the 395 overlapped eQTLs, 166 eSNPs mapped to 59 GWAS loci representing a total of 139 independent phenotypes. SNP rs705702 on chromosome 12 is a *cis*-eQTL for Ribosomal Protein S26 (*RPS26L*) chromosome 12 and is associated with PCOS. A full summary of overlapping loci is given in Table S22.

#### Summary data-based Mendelian randomisation (SMR) analysis with GWAS meta-analysis

Using summary statistics from the Sapkota *et al*.^23^ endometriosis meta-analysis, we tested for association between gene expression and endometriosis risk using the SNP as an instrumental variable in an SMR analysis. A single gene passed both the SMR and HEIDI tests, *LINC00339* (rs61768001, *p*_*SMR*_ = *4.82* × *10*^*−7*^) (Fig. S11). VEZT (rs7966079, *p*_*SMR*_ = *1.64* × *10*^*−4*^) sat just below the significance threshold and also passed the HEIDI test (Fig. S12). The HEIDI test is used to distinguish between effects due to the same causal SNP (pleiotropy) or distinct causal SNPs in linkage disequilibrium influencing the eQTL and genetic risk separately by testing heterogeneity in effect sizes of SNPs in the *cis*-eQTL region^34^. Several genes passed both the SMR and HEIDI tests for additional traits tested. These include *ATP13A1* with BMI, *ERAP*2 for both inflammatory bowel disease and celiac disease, *RPS*2*6* for both type 1 diabetes and rheumatoid arthritis, and *BTN2A1* with schizophrenia. The full list of significant genes and traits can be found in Table S23. All eQTLs for significant genes were also present in blood suggesting the effects are not tissue specific.

## Discussion

In this study, we analyzed genetic regulation of gene expression in endometrium in a large sample to increase the power for detection of eQTLs and analyze the overlap of eQTL signals with genomic regions associated with endometriosis and other reproductive traits. Methods for eQTL analysis generally restrict the data to probes/genes expressed in >90% of samples in a study. In endometrium, this excluded data for 9,626 probes mapping to 7,567 unique genes (39% of probes expressed in at least 1 sample). We analyzed this probe set separately and our results show significant differences in the proportions of women expressing many of these genes across the menstrual cycle and similar biological regulation to the genes showing quantitative changes in gene expression across the cycle. There was also evidence for genetic control of the expression for a small number of these genes.

We identified an additional 264 *cis*-eQTLs in 245 genes when compared to our previous analysis^35^ and replicated evidence of eQTLs for 187 of the 198 genes reported in the previous study^35^. We searched for eQTLs within regions of the genome associated with endometriosis risk identified from an independent study^23^. Two eQTLs overlap with known risk regions including eQTLs for *VEZT* and *LINC00339*^23^. *VEZT* was identified in a recent endometriosis meta-analysis as a potential casual gene from its association with an eQTL in blood, however, heterogeneity in the region suggested there was no single casual SNP in this instance^23^. In the current study, *VEZT* approached the SMR significance threshold and showed no evidence of heterogeneity suggesting that rs7966079 may contribute to both *VEZT* expression levels in the endometrium and endometriosis risk. Endometrial eQTLs have been identified for *LINC00339* previously^3,21^. Subsequent chromatin conformation capture experiments provided evidence for an interaction between endometriosis risk SNPs and the promoter of *LINC00339*^21,35^. *LINC00339* was identified as a potential causal gene passing the SMR test in this study with no evidence for heterogeneity in the region suggesting the same casual SNP regulates gene expression and the association with endometriosis.

We looked at overlap between *cis*-eQTLs in endometrium and trait associations from the GWAS catalogue. We observed overlap between 171 diseases or traits from the GWAS catalogue not reported previously^35^. Some eQTLs overlap with reproductive traits directly related to endometrial biology including endometrial cancer and PCOS. The GWAS SNP rs937213 at chromosome 5 associated with endometrial cancer^36^ is an eQTL for Signal Recognition Particle 14 (*SRP14*). *SRP14* is a ribonucleoprotein machine that controls the translation and intracellular sorting of membrane and secreted proteins^37^. The SNP rs705702, located on chromosome 12, and associated with PCOS risk^38^ is an eQTL for Ribosomal Protein S26 (*RPS26L*) suggesting *RPS26L* as a possible target transcript influencing *PCOS*^38^. *RPS26L* was shown to participate in a variety of cellular processes not directly associated with translation, such as p53 activity and endoplasmic reticulum (ER) stress^39,40^.

Approximately 68% of endometrial *cis*-eQTLs overlap with those identified in blood. Recent findings by the GTEx consortium suggest tissue specific eQTLs or eQTLs found in a limited number of tissues have greater regulatory effects^12^. The GTEx Project v6p data shows the average effect size of eQTLs decreases as the number of tissues in which they are present increases^41^. Our data support this hypothesis; the average effect size of endometrial eQTLs that are also present in blood is significantly smaller than the average effect size of endometrial eQTLs that are not present in blood.

Gene expression in the endometrium is strongly regulated with marked changes in the expression of many genes across the menstrual cycle. This variation is of two classes, changes in mean levels of expression for genes expressed in all samples and variation in the proportion women that express individual genes at different cycle stages. We observed significant variation in mean levels of expression for 32% of genes across the menstrual cycle in agreement with previous reports^1,2,7,42,43^. For probes expressed in only some samples, stage of the menstrual cycle significantly influenced the proportion of women expressing individual genes suggesting biological variation regulates both quantitative gene expression and the proportions of genes expressed or not expressed across the cycle. Our results show good agreement with genes recorded as “expressed/not expressed” in the Human Gene Expression Endometrial Receptivity database (HGex-ERdb)^41^ with 30–50% of genes overlapping between the two datasets depending on expression state and stage of the cycle (Table S24). Examples include *ANGPTL1*, encoding an angiopoietin-like protein expressed in different proportions of women across the cycle. It is a candidate for endometrial receptivity with a significant difference in expression reported between pre-receptive (early secretory) and receptive (mid-secretory) stages^41,44^. Another example is *OGDHL*, which is known to be transcribed in the proliferative stage and repressed in the secretory phase in the HGex-ERdb^41^.

Pathway analysis also provides strong support that the same complex biological changes occurring across the cycle drive changes in both the mean expression of many widely-expressed genes, and the expression/non-expression of genes in different proportions of individuals at different times of the cycle. The most highly enriched hallmark pathways from both the significant differentially expressed gene set and expressed/non-expressed gene set were related to endometrial biology and included “oestrogen early and late response”, “epithelial mesenchymal transition”, and “kras signalling”. Oestrogen is one of the main hormones regulating endometrial cell proliferation. Studies have shown increased proliferation of the luminal and glandular epithelium and stromal cells during the proliferative phase is mediated by an increase in oestrogen and expression of oestrogen receptors 1 and 2 in both epithelial and stromal cells^45–47^. Changes in gene expression in response to oestrogen have also been reported, as have changes in gene expression across the cycle that coincides with changes in oestrogen levels^2,35,43,48,49^. Enrichment of the “oestrogen early and late response” pathways in both gene sets suggest that both transcription levels and activation are partially driven by changes in oestrogen levels and response to these changes. We found no evidence for interactions between stage of the cycle and genotype effects on gene expression (context-specific eQTLs). The lack of replication of context-specific effects^35^ reported previously may be due to the more stringent inclusion criteria for samples in the current study and the increased sample size in the secretory phase of the cycle.

We analyzed results for differences between endometriosis cases and controls in the combined data. In the RWH dataset, diagnosis of endometriosis was made at laparoscopy, but in the IVF dataset, endometriosis diagnosis was by self-report. After correcting for stage of the menstrual cycle, some genes/probes showed nominal evidence of differences between endometriosis cases and controls. However, there were no significant effects of endometriosis on mean differences in gene expression, or transcriptional silencing, in the eutopic endometrium following Bonferroni correction for multiple testing or the less stringent FDR correction. This was also true when the analysis was restricted to the RWH dataset where presence or absence of endometriosis was confirmed at laparoscopy. Differences between endometriosis cases and controls have been reported previously^7,32^, although many of these are based on small sample sizes and our results in the larger sample set, corrected for stage of the cycle and multiple testing, did not replicate previous reports.

The most significant new eQTLs detected include eQTLs for *NEDD8, RPS26, SNHG17, SNHG*5 and *WARS2*. *NEDD8* (neural precursor cell expressed, developmentally down-regulated 8) is a ubiquitin-like protein that targets the ubiquitin E3 ligase family^50^ and may be important in regulating normal endometrial function^51^. One study found *NEDD8* was expressed in luminal epithelium, glandular epithelium and the stromal cells during the menstrual cycle and that, when inhibited, it significantly decreased proliferation in human endometrial stromal cell lines (HESC) and disrupted decidual transformation^51^. A previous study on the association between endometrial eQTLs, detected in endometrial cells from mid-luteal phase, and fecundity in women, identified 423 *cis*-eQTLs for 132 genes^52^. We detected eQTLs for 68 of the genes identified by Burrows *et al*.^52^. eQTLs for the two genes associated with fecundability, *TAP2* and *HLA-F*, were not replicated in our analysis, however eSNP rs2523393 previously associated with *HLA-F* expression and fecundability was associated with *HLA-H* expression in our analysis supporting a potential role of *HLA-H* in female fertility. We have compared our results with biomarkers for endometrial receptivity and a recent meta-analysis of transcriptomic biomarkers. We identified eQTLs in 7 of the 57 including *PAEP, SPP1, IL15, TSPAN8, OLFM1, MMP7* and *CXXC1*^53^. The direction of effect was consistent with that reported by Altmäe *et al*.^53^ *PAEP* is important in regulating the endometrial environment for implantation; changes in expression of this gene have been associated with implantation failure^54,55^ and it has a suggested role in anti-inflammatory response during the window of implantation^55^. *IL15* is a cytokine expressed in both human endometrial stromal and epithelial cells. It is involved in immune regulation through the stimulation and regulation of natural killer cell proliferation and has a role in decidualisation^56,57^. *IL15* has also been shown to stimulate proliferation and invasion of endometrial stromal cells in ectopic endometrium of women with endometriosis^58^. Similarly we capture changes in expression of 19/22 genes defined as biochemical pregnancy biomarkers and detect eQTLs for three markers, *CDC2, MFAP2* and *OLFM1*^59^. CDC2 is important for cell cycle regulation and endometrial stromal cell proliferation^60,61^. Decreased expression of MFAP2 has been observed in women with multiple implantation failures^62^. Genetic regulation of *PAEP, IL15, CDC2* and other genes may be an important consideration when using these as biomarkers and for the understanding of potential mechanisms behind reproductive disorders.

We identified 3366 *cis*-eSNPs regulate expression of 41 transcription factors. The SNP rs4970988 at chromosome 1 displayed a strong cis-association with Aryl Hydrocarbon Receptor Nuclear Translocator (*ARNT*), encoding the transcription factor ARNT. *ARNT* encodes a protein that binds to ligand bound Aryl Hydrocarbon receptor and promotes xenobiotic metabolism^63^ and Caspase Recruitment Domain Family Member 8 (*CARD8*) that negatively regulates IL1B secretion^64^ and apoptosis^65^. *ARNT* is expressed widely across reproductive tissues e.g. uterus and ovary (GTEx) with expression changes in some gynecological pathologies such as uterine leiomyomata^66^.

The increase in sample size provided greater power to detect additional *cis*-eQTLs and the first evidence of *trans*-eQTLs in endometrium. We identified 1,593 significant *trans*-eQTLs. eSNPs with both *cis* and *trans*-genes suggest a shared mechanism of regulation as demonstrated by the GTEx consortium where Mendelian Randomisation analysis measuring the causal impact of *cis*-genes on *trans*-genes found strong evidence for regulation of *trans*-genes by the *cis*-gene^12^. SNP rs4958465 and rs117677211 are *cis*-eQTL for *SPARC* and *ITGB1* respectively and for several *trans*-genes within the endometrium. Both *SPARC* and *ITGB1* have been associated with endometrial biology previously^25–31^. *SPARC* is a matrix-associated protein involved in collagen binding and deposition and extracellular matrix assembly, cellular adhesion, angiogenesis, migration, proliferation, tissue remodelling^25–27^. *SPARC* has been a gene of interest in multiple endometrial disease pathologies including endometriosis where it has been reported as deregulated in endometriotic lesions in women with endometriosis^28^. *SPARC* is also overexpressed in endometrial cancer stem-like cells^29^. *ITGB1* has been reported as deregulated in endometrial disease with increased expression of *ITGB1* detected in a small number of endometrial samples from women with endometriosis compared to women without the disease^30^. Downregulation of miR-183, a negative regulator of *ITGB1*, in ectopic and eutopic endometrial tissues has been shown to increase levels of *ITGB1*, which is hypothesised to promote adhesion and invasiveness of endometrial stromal cells^30,31^.

Whilst new evidence suggests that <4% of *trans*-eQTLs are shared between tissues and *trans*-eQTLs are predominantly tissue specific^12^, we identified a *trans*-eQTL located on chromosome 12 that has been identified previously in CD4^+^ and CD8^+^ T cells. Of note, 50% of the *trans*-genes identified in our study also replicated in T cells^67^. The sentinel SNP rs1131017 located in the 5’UTR of ribosomal protein S26 (*RPS26*) is reportedly in LD with risk SNPs for Type 1 diabetes (T1D)^67–70^, vitiligo^67,71^, PCOS^38,67^ and rheumatoid arthritis^67,72^. We confirmed overlap with risk regions in T1D and rheumatoid arthritis using SMR analysis which found the *RPS26* endometrial *cis*-eQTL expression levels were associated with risk SNPs for T1D and rheumatoid arthritis, the gene passing both the SMR and HEIDI test suggesting a causal relationship.

Our study has several limitations. Endometrial samples were collected from women attending clinics for pelvic pain and endometriosis, or for IVF treatment. This is a limitation, but difficult to avoid given the issues of collecting biopsies from a community sample of women not attending clinics. The presence of endometriosis was recorded at laparoscopy (RWH clinics) or from self-report (IVF clinics). Medical records were reviewed for the participants and any gynaecological conditions were noted and recorded. Our selection criteria excluded women who had abnormal endometrial histopathology, who were on hormonal treatment, or of non-European ancestry. Careful comparison of results from women recruited in the endometriosis or IVF clinics showed very little difference in endometrial gene expression between the groups. We had limited data on other gynaecological conditions in our dataset, but post-hoc studies suggested no evidence of confounding of our results. Stage of the menstrual cycle has the strongest effect on gene expression in the endometrium and comparisons of our results show good replication with published data. We also show excellent replication with previous eQTL studies in endometrium. The lack of differences in gene expression between the two groups with different ascertainment and good replication of other published results suggest any limitations in recruiting patients attending clinics has not influenced the results or conclusions.

Another limitation is the tissue is made up of multiple cell types and there are changes in cellular composition and cell activity across the cycle. Statistical methods have been developed to predict cell count in whole blood without cell sorting, but this requires a very large number of samples. Single cell RNA-seq methods may overcome some of these limitations in the future.

In conclusion, we identified *cis*-eQTLs for 417 genes in endometrium. Two *cis*-eQTLs overlap genomic regions associated with endometriosis with good evidence for the causal SNP in each region influencing endometriosis risk and the expression of *LINC00339* on chromosome 1 or expression of *VEZT* on chromosome 12. The results provide stronger support for effects of the endometriosis risk variant(s) increasing *VEZT* expression in the endometrium. We did not detect novel endometrial eQTLs in the 12 other regions associated with endometriosis and further studies will be needed to understand the functional effects of these genetic risk factors. The eQTL analysis in endometrium may be relevant to other reproductive traits and we identified one novel *cis*-eQTL located in a genomic region associated with PCOS. Analysis of gene expression in the endometrium shows strong regulation across the menstrual cycle for both quantitative changes in expression and in the frequency of detecting expression of individual genes. The genetic effects on endometrial gene expression identified both *cis-* and *trans*-eQTLs with potential roles in endometrial biology, including several genes implicated in endometrial receptivity where the eQTLs might complicate their role as biomarkers.

## Methods

### Sample collection

We recruited 229 women of European ancestry attending clinics at the Royal Women’s Hospital or Melbourne IVF in Melbourne, Australia. Ethical approval for the study was obtained from the Royal Women’s Hospital Human Research Ethics Committee (Projects 11–24 and 16–43), and the Melbourne IVF Human Research Ethics Committee (Project 05-11). Informed consent was obtained from all participants and all methods were performed in accordance with institutional approved guidelines and regulations. Group 1 (RWH patients, *n* = 165) were reproductive-aged women who underwent laparoscopic surgery for investigation of pelvic pain and/or endometriosis. Detailed patient questionnaires, past and present clinical histories, pathology results and surgical notes were recorded for each participant. For the RWH dataset, endometrial tissue samples were collected by curettage from women at the time of surgery. A blood sample was collected from all patients prior to surgery. All RWH subjects were free from exogenous hormone treatment in the three months prior to surgery. A diagnosis of endometriosis was made by the surgeons following visual inspection at laparoscopy; 112 women had a positive diagnosis of endometriosis (Table 7). We recorded other gynecological co-morbidities where these were noted in the clinical records. Of patients who received an ultrasound; 10/61 patients had a diagnosis of uterine fibroids and 16/59 patients had a diagnosis of adenomyosis (Table 7).

**Table 7:** Clinical details of subjects.

	Group 1 (RWH)	Group 2 (IVF)
*Number of samples*	165	64
*Age* (*years* ± *SEM*)	31.21 ± 0.53	36.56 ± 0.51
***Endometriosis***		
*Diagnosis methods*	Surgically confirmed	Self-report
*Diagnosis*		
*Yes*	67.9% (112/165)	32.8% (21/64)
*No*	29.1% (48/165)	64.1% (41/64)
*Unknown*	3.0% (5/165)	3.1% (2/64)
***Uterine fibroids***		
*Diagnosis*		
*Yes*	6.1% (10/165)	
*No*	30.9% (51/165)	
*Unknown*	63.0% (104/165)	100% (64/64)
***Adenomyosis***		
*Diagnosis*		
*Yes*	9.7% (16/165)	
*No*	26.1% (43/165)	
*Unknown*	64.2% (106/165)	100% (64/64)
***Histological cycle staging***		
*Menstrual (M)*	6.7% (11/165)	0% (0/64)
*Early proliferative (EP)*	3.0% (5/165)	0% (0/64)
*Mid proliferative (MP)*	39.4% (65/165)	6.3% (4/64)
*Late proliferative (LP)*	9.7% (16/165)	6.3% (4/64)
*Early secretory (ES)*	9.7% (16/165)	53.0% (34/64)
*Mid secretory (MS)*	17.6% (29/165)	34.4% (22/64)
*Late secretory (LS)*	13.9% (23/165)	0% (0/64)

Group 2 (IVF patients, *n* = 64) were reproductive-aged women undertaking IVF who consented to undertake a tracking cycle with a mid-luteal phase Pipelle endometrial biopsy. For the IVF group, the time of ovulation was estimated by detection of the LH surge using urinary LH detection kits, with an outpatient Pipelle endometrial biopsy 5–7 days after ovulation. A peripheral blood sample was also collected at the time of biopsy. IVF subjects were not receiving exogenous hormones during their tracking cycle, but 29 IVF patients received ovarian stimulation as part of an IVF treatment cycle one month prior to biopsy. Self-reported information on endometriosis (*n* = 21) was collected for the IVF group.

For both sample groups, endometrial tissue samples were split and either stored in RNA*later* (Life Technologies, Grand Island, NY, USA) at −80 °C until RNA extraction, or formalin fixed and processed routinely for histological assessment. Histological sections from all biopsy samples were viewed by an experienced pathologist and endometrial cycle stage was determined (Menstrual (M) = 11, Early Proliferative (EP) = 5, Mid-Proliferative (MP) = 69, Late Proliferative (LP) = 20, Early Secretory (ES) = 50, Mid-Secretory (MS) = 51 and Late Secretory (LS) = 23).

We included samples if their histological stage of menstrual cycle could be assigned to one of the seven stages and we could obtain good quality RNA from the samples. Individuals were excluded from further analysis if samples showed any sign of abnormality or their histological stage of menstrual cycle could not be determined. Neither group were taking hormones in the cycle when the endometrial biopsies were taken.

The study was approved by the Human Research Ethics Committees of the Royal Women’s Hospital, Melbourne, the QIMR Berghofer Medical Research Institute and The University of Queensland and all women gave written consent.

### RNA extraction

Total RNA was extracted from homogenized endometrial tissues using RNA lysis solution (RLT buffer) and AllPrep DNA/RNA mini kit according to the manufacturer’s instructions (QIAGEN, Valencia, CA). RNA integrity was assessed with the Agilent Bioanlayzer 2100 (Agilent Technologies, Santa Clara, CA) with all samples having high-quality RNA (RNA Integrity Number (RIN) >8), and concentrations were determined using the NanoDropND-6000.

### Gene expression array

Total RNA was amplified and converted to biotinylated cRNA using Ambion Illumina TotalPrep RNA amplification kit (Ambion). Expression profiles were generated by hybridising 750 ng of cRNA to Illumina Human HT-12 v4.0 Beadchips (Illumina Inc, San Diego, USA) as described previously^3^. Samples were scanned using an Illumina iScan Reader. Samples were randomised across arrays and array positions.

### Genotyping

Whole blood DNA samples were genotyped on HumanCoreExome chips and Infinium PsychArray (Illumina Inc, San Diego). Quality control of genotypes was performed using the program PLINK^73^. SNPs with a missing rate of >5% (–geno 0.05 command), MAF < 1 × 10^−4^ (–maf 0.0005 command) and with Hardy-Weinberg Equilibrium (HWE) p < 1 × 10^−6^ (–hwe 0.000001 command) were removed leaving 282,625 SNPs for imputation. Imputation was performed using the 1000 Genomes Phase 3 V5 and was phased using ShapeIt V2 on the Michigan Imputation Server^74^. Following imputation SNPs with low MAF < 0.05 and poor imputation quality were removed (R^2^ < 0.08) leaving 6,004,543 autosomal SNPs for analysis.

### Gene expression normalisation

The following normalisation procedures were applied to the raw expression data for analysis as described previously^3^. Briefly, pre-processing of data generated by the Illumina iScan Reader was carried out using Illumina GenomeStudio software (Illumina Inc., San Diego). Any probe with a detection p-value provided by GenomeStudio greater than 0.05 was considered as not expressed for that given sample.

To achieve a stabilized distribution across average expression levels, pre-processed transcript levels were transformed using a quantile adjustment across individuals, followed by scaling to log2. Further normalisation was performed to allow expression levels to be compared across chips and genes.

### Differential expression

We sought to evaluate changes in gene expression across menstrual stages. To avoid biasing our results with genes that were not expressed at certain stages of the menstrual cycle, we restricted our analysis to only those genes that were expressed in ≥90% of samples, leaving 15,262 probes, mapping to 12,321 unique RefSeq genes (Fig. 1). We performed the differential expression analysis between stages of the menstrual cycle as described previously^3^. Briefly, EP and MP samples were combined with the LP samples as proliferative (P) group (*n* = 94), and comparisons were made across successive cycle stages: M vs.P; P vs. ES; ES vs. MS and MS vs. LS, using the *eBayes* method, which is implemented in the limma package. The resulting p-values were corrected for multiple testing to control the false discovery rate (FDR) using the Benjamini-Hochberg method. We selected probes with a fold change >1.5 (corresponding to a 1.5 standard deviations) and a study-wide FDR < 0.05 as differentially expressed.

### Expressed or not expressed genes

To identify genes activated or repressed during different stages of the menstrual cycle and between cases and controls, probes were classified as not expressed in samples (repressed) if they had a detection p-value greater than 0.05, all other probes with p-values less than or equal to 0.05 were classified as expressed (activated). Expressed/not expressed status was set as a binary dependant variable for each of the 229 samples at each of the probes. Probes expressed in ≥90% of samples and probes expressed in no samples were excluded from the analysis, 9,626 probes remained (Fig. 1). The difference between the proportion of genes activated or repressed between menstrual (M) and the combined proliferative stage, consisting of EP, MP and LP stages was identified by performing logistic regression analysis on samples using the following model - equation (1):

1 $${ln}(\frac{\hat{{p}}}{1-\hat{{p}}})={{\beta }}_{0}+{{\beta }}_{1}\ast {stage}+{{\beta }}_{2}\ast {disease}+{{\beta }}_{3}\ast {proportion}$$

where $\hat{{\rm{p}}}$ denotes the probability that the probe is expressed and $1-\hat{p}$ the probability that the probe is not expressed, β_0_ the intercept, β_1_ is the regression coefficient of the stage of cycle, β_2_ is the regression coefficient of the disease status and β_3_ is the regression coefficient of the proportion of all probes expressed in each sample as a measure of sample quality. The analysis was repeated for successive cycle stages, P vs. ES, ES vs. MS and MS vs. LS. To correct for multiple testing an FDR cut-off 0.05 was applied to the resulting p-values using the Benjamini-Hochberg method.

### Pathway analysis

Pathway analysis was conducted using the “GENE2FUNC” function at FUMA GWAS web-based platform^75^. Gene lists examined included those identified from the differential expression analysis and the ‘activated/repressed’ analysis. The p-values were adjusted using the Benjamini-Hochberg (FDR) multiple correction method. A pathway was considered significant at the *p* < 0.05 threshold.

### Endometriosis case/control analysis

A differential expression analysis was also used to test for any differences in expression levels of probes expressed in ≥90% of samples between cases and controls. The *eBayes* method in limma was again used, this time correcting for stage of cycle. Differences in gene expressed or not expressed between cases and controls was also tested using the logistic regression model explained previously with the exception of adjusting for stage of cycle in place of disease status. Resulting p-values were corrected for multiple testing and significance thresholds applied, as outlined in the previous differential expression and gene activation analyses.

### eQTL analysis

An eQTL analysis was performed on 229 individuals of European ancestry. A total of 15,262 probes mapping to 12,321 unique genes and expressed in 90% of samples were included in the analysis. Restricting the eQTL analysis to probes expressed in 90% of samples is common practice in eQTL studies. In order to minimize bias between stages of the cycle and have sufficient power (~80%) to detect eQTLs at an FDR < 0.05 at SNPs with low minor allele frequency, a sample size of at least 200 is necessary. In addition, relaxing this threshold below 90% introduces false positive results for eQTLs. We tested for any association between normalised expression levels at each probe with SNP genotypes using a linear regression model in the program PLINK (−linear command)^73^. Disease status and stage of cycle were fitted as covariates in the model. *Cis*-eQTls were subsequently annotated in the output and defined as eQTLs in which the associated SNP was located +/−250 kb from the probe starting position. *Trans*-eQTLs were defined as eQTLs between SNPs and a probe on a different chromosome. We performed conditional analysis on both sentinel *cis*-eQTL’s which met a study-wide significance threshold of *p* < 3.3 × 10^−9^ and those that met an FDR cut-off of <0.05, to identify any secondary independent eQTLs.

### Functional annotation

Using previously identified ESR binding sites mapped by Carroll *et al*.^49^ we tested for overlap between sentinel eSNPs for *cis* and *trans*-eQTLs and ESR binding sites. We also tested for any overlap between the region surrounding (±50 kb) the transcription start site (TSS) of genes significantly differentially expressed or expressed/not expressed across the cycle and ESR binding sites. All three gene sets, genes with eQTLs, genes significantly differentially expressed across the menstrual cycle and expressed/not expressed genes across the menstrual were also annotated against known transcription factors using the data by Vaquerizas *et al*.^76^.

### eBTL analysis

A new approach to identifying the effect of genotype on the proportion of samples expressing a probe was implemented in this study. We performed an “expression binary trait loci” analysis in which probe expression was treated as a binary trait, probes expressed at any level in a given sample were classified as “expressed” or “activated” and if not expressed in a given sample were classified as “not expressed” or “repressed”. The eBTL was performed on the same 229 individuals using the 9,626 variably expressed probes. Using logistic regression in PLINK (−logistic command) we tested for any association between a probe being expressed versus not expressed and SNP genotypes. Like the eQTL analysis both disease status and stage of cycle were included as covariates in the model. Associated SNPs within 250 kb of the probe starting position were defined as *cis* and those located on different chromosomes were defined as *trans*. A genome-wide significance threshold of *p* < 5.2 × 10^−9^ was applied along with a less stringent FDR cut-off of <0.05.

### Context specific eQTL analysis and overlap of eQTLs with differentially expressed genes

To investigate the relationship between genes differentially expressed across the cycle and eQTLs we tested for overlap between the two probe sets and calculated a chi-square statistic to determine if this overlap deviates from what is expected. Using only eQTL’s passing the Bonferroni correction and applying the method outlined by Fung *et al*.^35^ we tested for any interaction between the genotype of an individual and stage of cycle on the expression of 125 *cis*-eQTLs corresponding to genes differentially expressed between different stages of the menstrual cycle. Genes differentially expressed between menstrual and the three collective proliferative (P) phases, P vs. ES, ES vs. MS and MS vs. LS were tested.

### Overlap between endometrial and blood eQTLs

Blood eQTLs from the Consortium for the Architecture of Gene expression (CAGE) dataset^77^, consisting of 11,204 *cis*-eQTLs identified across 2,765 individuals, were used to determine overlap with independent endometrial eQTLs. Additional eQTLs identified in blood (*n* = 338), were downloaded from GTEx to determine overlap with endometrial eQTLs. eQTLs overlapped if they had the same probe and associated SNP or if the SNP associated with the probe in the CAGE/GTEx dataset had a minimum linkage disequilibrium (LD) *r*^*2*^ > 0.7 with the endometrial SNP based on the 1000 Genome phase 3 reference panel.

### Overlap with variants associated with other traits and diseases

#### GWAS catalogue trait

Trait-associated GWAS SNPs were downloaded in June 2017 from the NHGRI Catalog of Published GWAS using the default p-value threshold of 5 × 10^−8^. The degree of overlap between endometrial tissue eQTLs and GWAS loci were based upon a minimum LD *r*^*2*^ > 0.7 between the eSNP and GWAS SNP in the 1000 Genome reference panel. SNPs that were not identified in populations of European descent were excluded.

#### Summary data-based Mendelian randomisation (SMR) analysis with GWAS meta-analysis

SMR analysis^34^ was used to identify causal genes with expression levels associated with endometriosis by pleiotropy. We conducted the SMR using GWA meta-analysis summary data from Sapkota *et al*.^23^ consisting of >12,000 European endometriosis cases and 7,899,416 SNPs alongside the endometrial eQTL data generated in this study. A total of 453 eQTL probes that reached Bonferroni genome-wide significance were included in the analysis and an SMR p-value threshold of 1.1 × 10^−4^ (0.05/453 probes) was applied to determine SMR genome-wide significance. A HEIDI (heterogeneity in dependent instruments) test, incorporated in the SMR software package, was also applied to test heterogeneity of effect sizes in *cis*-eQTL regions. A p-value of <0.05/m_SMR_sig, where m_SMR_sig is the number of probes that passed the genome-wide SMR threshold, suggested heterogeneity in the effect values estimated for SNPs in the region and the possibility on an association due to colocalisation and LD between multiple casual SNPs rather than pleiotropy.

SMR analyses were also performed using endometrial eQTLs and several GWAS summary datasets including BMI, body fat percentage, leptin, lipid levels including HDL, LDL, TC and TG, coronary artery disease, heart rate, rheumatoid arthritis, celiac disease, inflammatory bowel disease, ulcerative colitis, type 1 diabetes, type 2 diabetes, glucose levels, insulin levels, ADHD, alzheimer’s, schizophrenia, bipolar disorder, major depressive disorder, autism, motor neurone disease, age-related macular degeneration and osteoporosis.

### Ethics approval and consent to participate

The study was approved by the Royal Women’s Hospital Human Research Ethics Committee (Projects 11–24 and 16–43), and the Melbourne IVF Human Research Ethics Committee (Project 05-11) and the University of Queensland. Informed consent was obtained from all participants.

### Availability of data and materials

All eQTL data are available at http://reproductivegenomics.com.au/shiny/eeqtl2/. Other data generated during this study are included in this article and its supplementary information files.

## Electronic supplementary material

Dataset1

Dataset 2
