# Supplementary material for: Genetic regulation of disease risk and endometrial gene expression highlights potential target genes for endometriosis and polycystic ovarian syndrome
Source: Sci Rep. 2018 Jul 30;8:11424. doi: 10.1038/s41598-018-29462-y (PMC6065421; doi:10.1038/s41598-018-29462-y)
Supplement: Supplementary file 1 — Dataset1 [file 41598_2018_29462_MOESM1_ESM.docx]

**Supplementary Figures**

**Genetic regulation of disease risk and endometrial gene expression highlights potential target genes for endometriosis and polycystic ovarian syndrome**

Jenny N. Fung ^1, *, +^, Sally Mortlock ^1, *^_,_ Jane E. Girling ^2^, Sarah J. Holdsworth-Carson ^2^, Wan Tinn Teh ^2^, Zhihong Zhu ^1^, Samuel W. Lukowski ^1^, Brett D. McKinnon ^1,3^, Allan McRae ^1^, Jian Yang ^1^_,_ Martin Healey ^2^, Joseph E. Powell ^1^_,_ Peter A.W. Rogers ^2^ and Grant W. Montgomery ^1^

1. The Institute for Molecular Bioscience, The University of Queensland, Brisbane, QLD 4072, Australia

2. Gynaecology Research Centre, The University of Melbourne, Department of Obstetrics and Gynaecology, Royal Women’s Hospital, Parkville VIC 3052, Australia

3. Department of Obstetrics and Gynaecology, University hospital of Berne, 3010, Berne, Switzerland

4. Department of Anatomy, University of Otago, Dunedin, New Zealand

* These authors contributed equally to this study

**+ Corresponding author:** Jenny N. Fung, Institute for Molecular Bioscience, The University of Queensland, 306 Carmody Road, Building 80, St Lucia QLD 4072 Australia; Email: [j.fung1@uq.edu.au](mailto:j.fung1@uq.edu.au); Phone +617 3346 2394

_
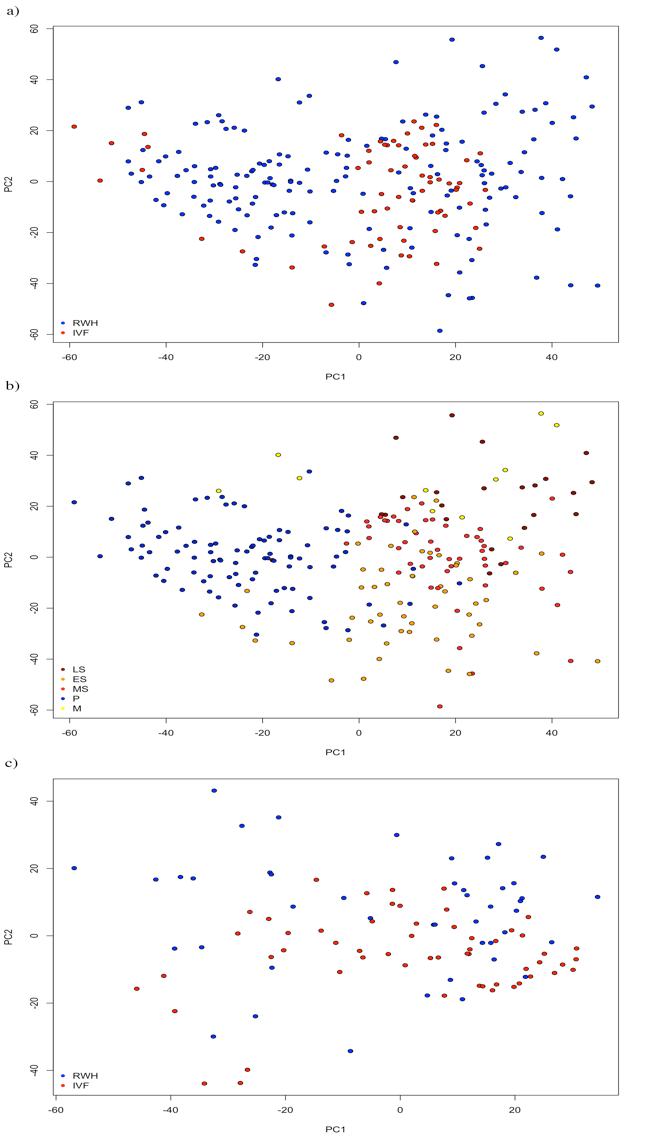
_

Figure S1. a) PCA plot of 229 samples using 100% expressed probes. IVF dataset was shown in red and the RWH dataset was shown in blue. b) PCA plot of 229 samples using 100% expressed probes. Samples from the menstrual phase (M) shown in yellow, proliferative phase (P) shown in blue, early-secretory (ES) shown in orange, mid-secretory (MS) shown in red and late-secretory (LS) shown in brown. c) PCA plot of 101 samples from the early and mid-secretory cycle phases using 100% expressed probes. IVF dataset was shown in red and the RWH dataset was shown in blue.

­­­­­­­


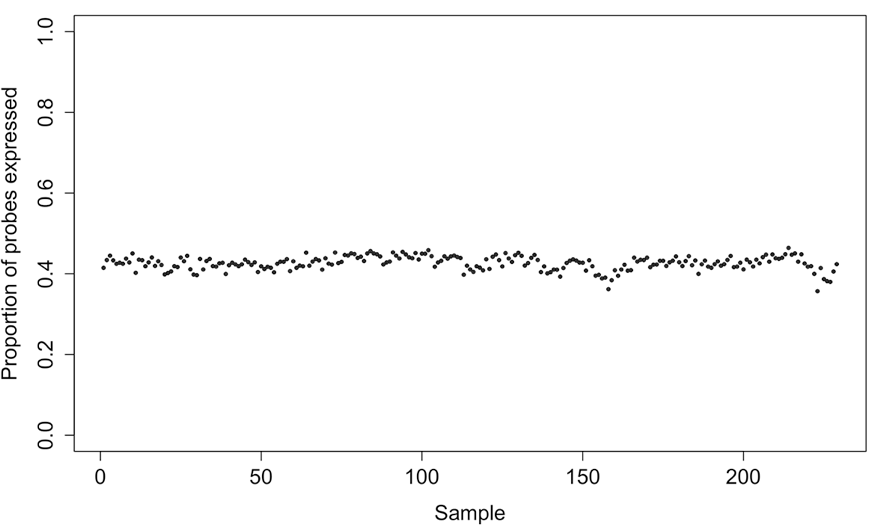


Figure S2. Proportion of total probes (*n=*47,150) expressed in each of the 229 endometrial samples.


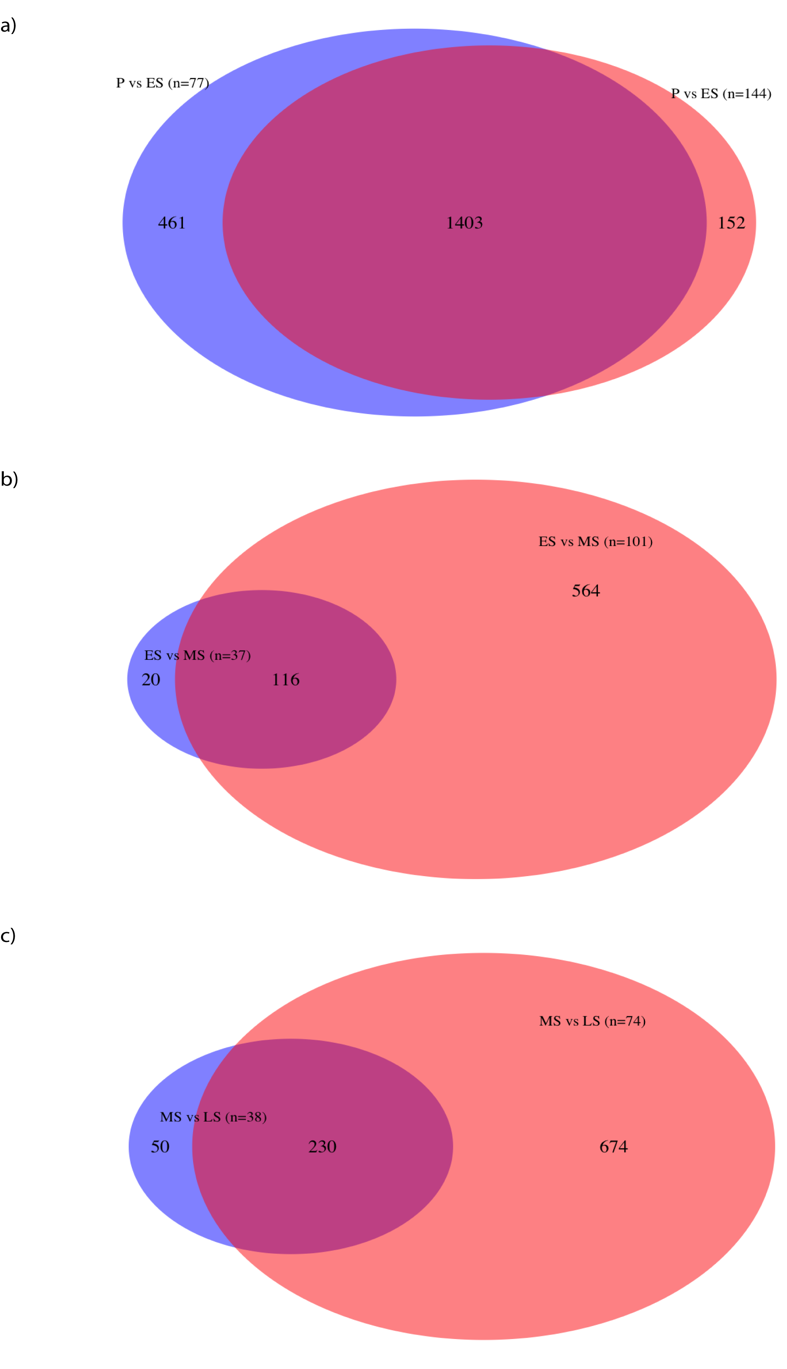


Figure S3. The Venn diagrams showing the number of regulated genes across the menstrual cycle and the number of genes shared between the previous study (Fung *et al*., 2017 ; blue) and this study (red).


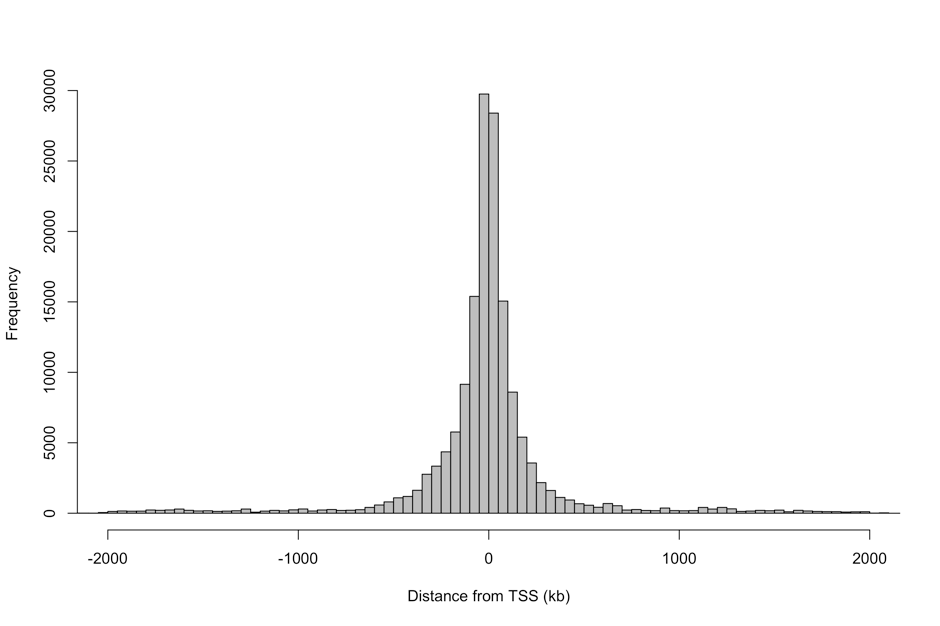


Figure S4. Distribution of the distance between significant eQTL eSNPs and the transcription start site (TSS) of the gene for which they are associated.


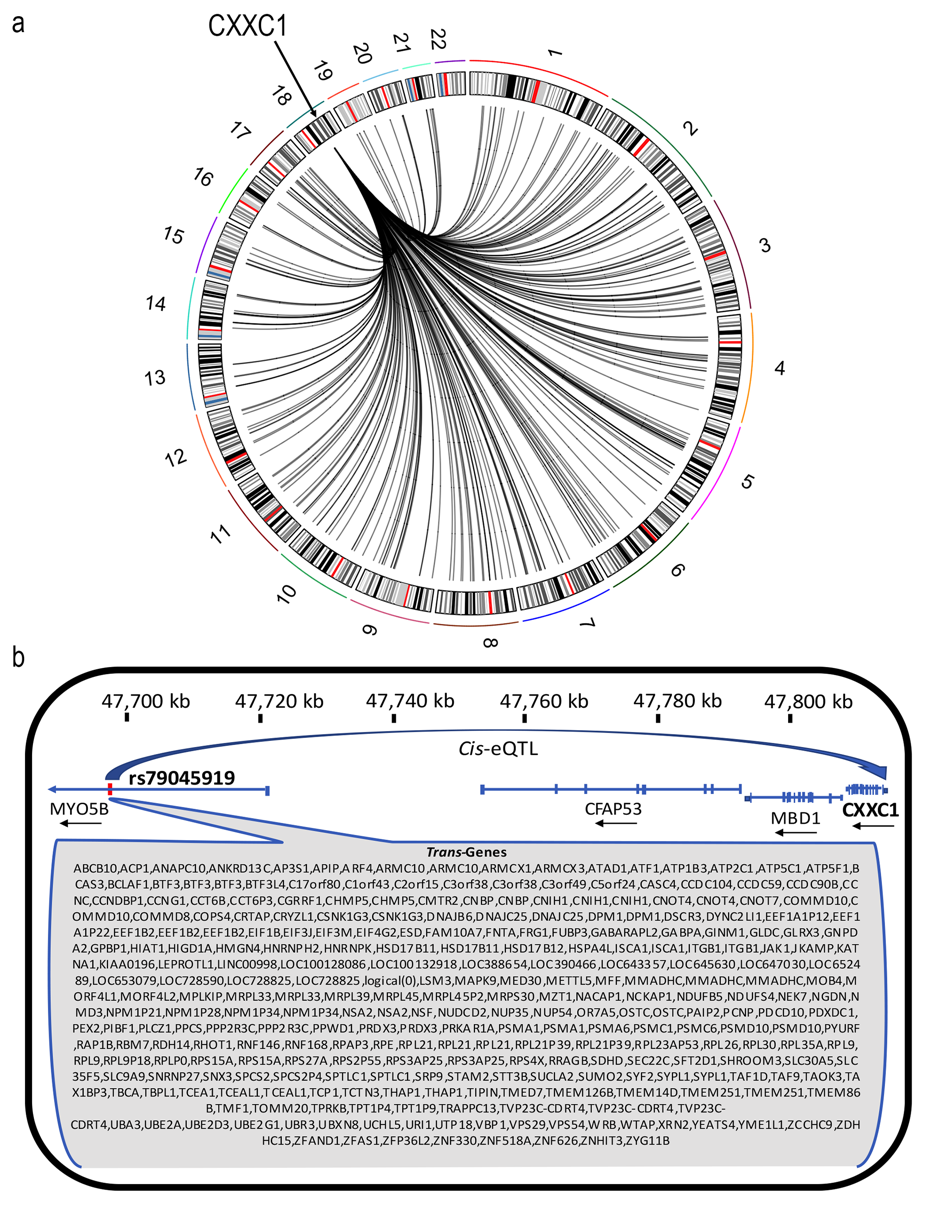


Figure S5. a) Circos plot showing the position of a *cis*-eQTL between rs79045919 and CXXC1 and the 269 *trans*-eQTLs with the same SNP. b) rs79045919-CXXC1 *cis*-eQTL on chromosome 18 and the genes that it effects in *trans*.


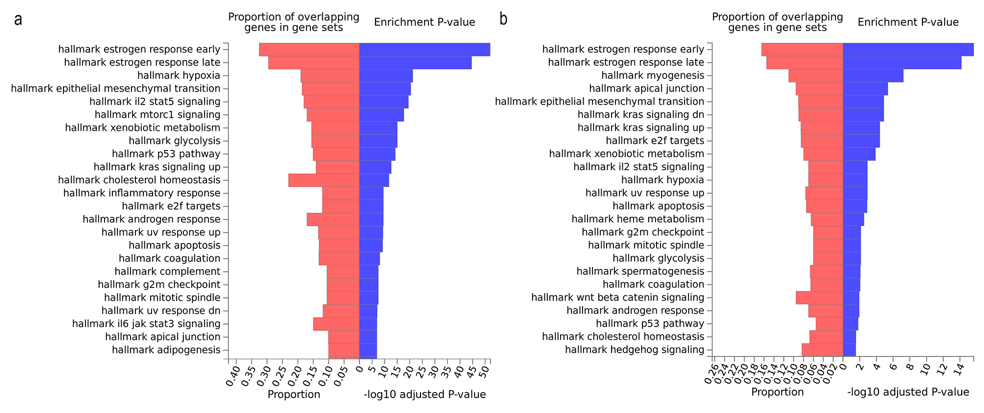


Figure S6. a) Pathways enriched for genes significantly differentially expressed across the menstrual cycle with ESR binding sites within 50kb of their TSS and b) pathways enriched for significant on/off genes across the cycle with ESR binding sites within 50kb of their TSS.


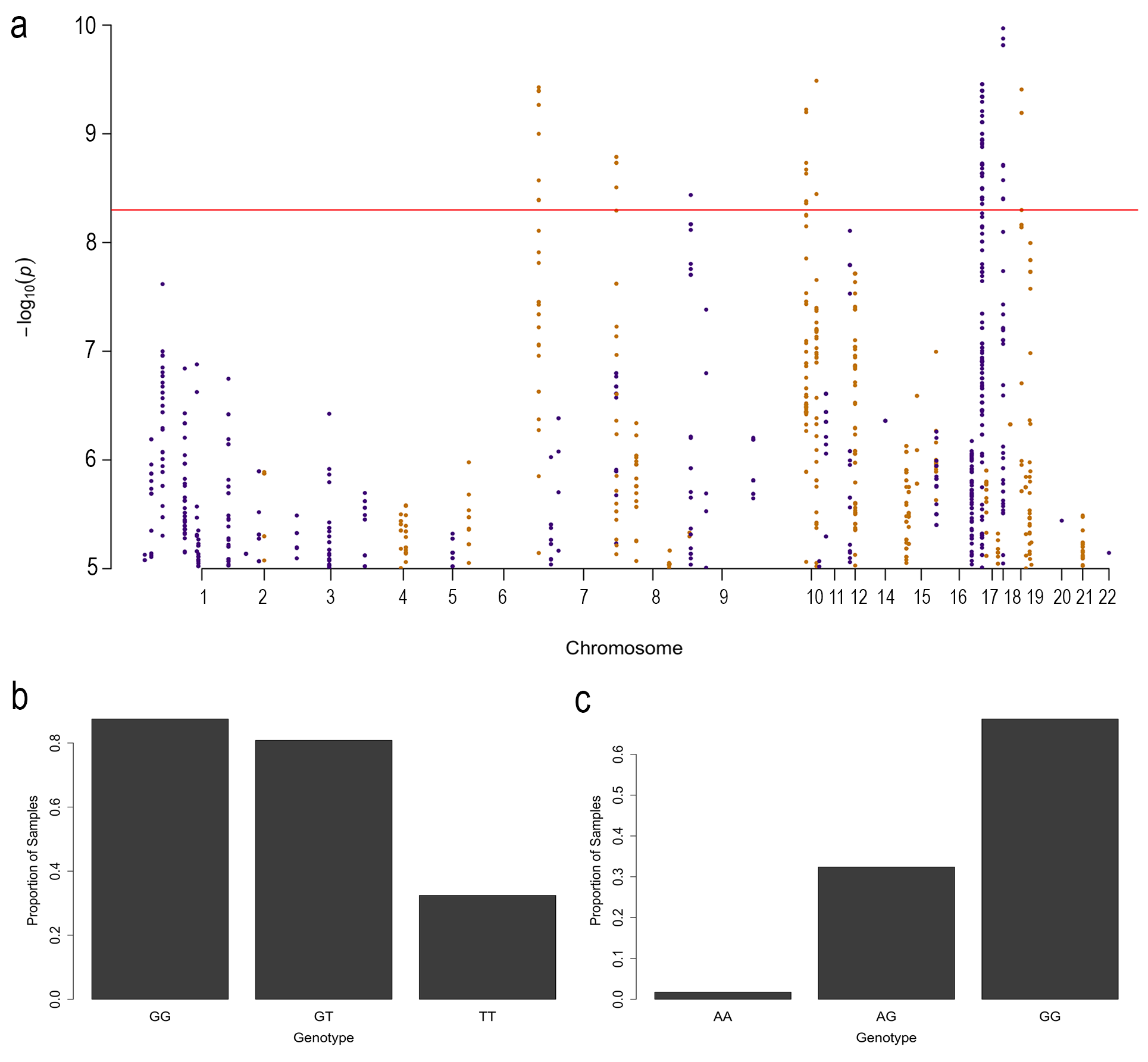


Figure S7. a) Manhattan plot of top SNPs associated with the proportion of samples expressing each probe. Each point represents a SNP and the red line represents a Bonferroni threshold of 5.2x10^-9^. b) Proportion of samples with different genotypes at rs10411704 expressing MAG. c) Proportion of samples with different genotypes at rs627262 expressing VAPA.


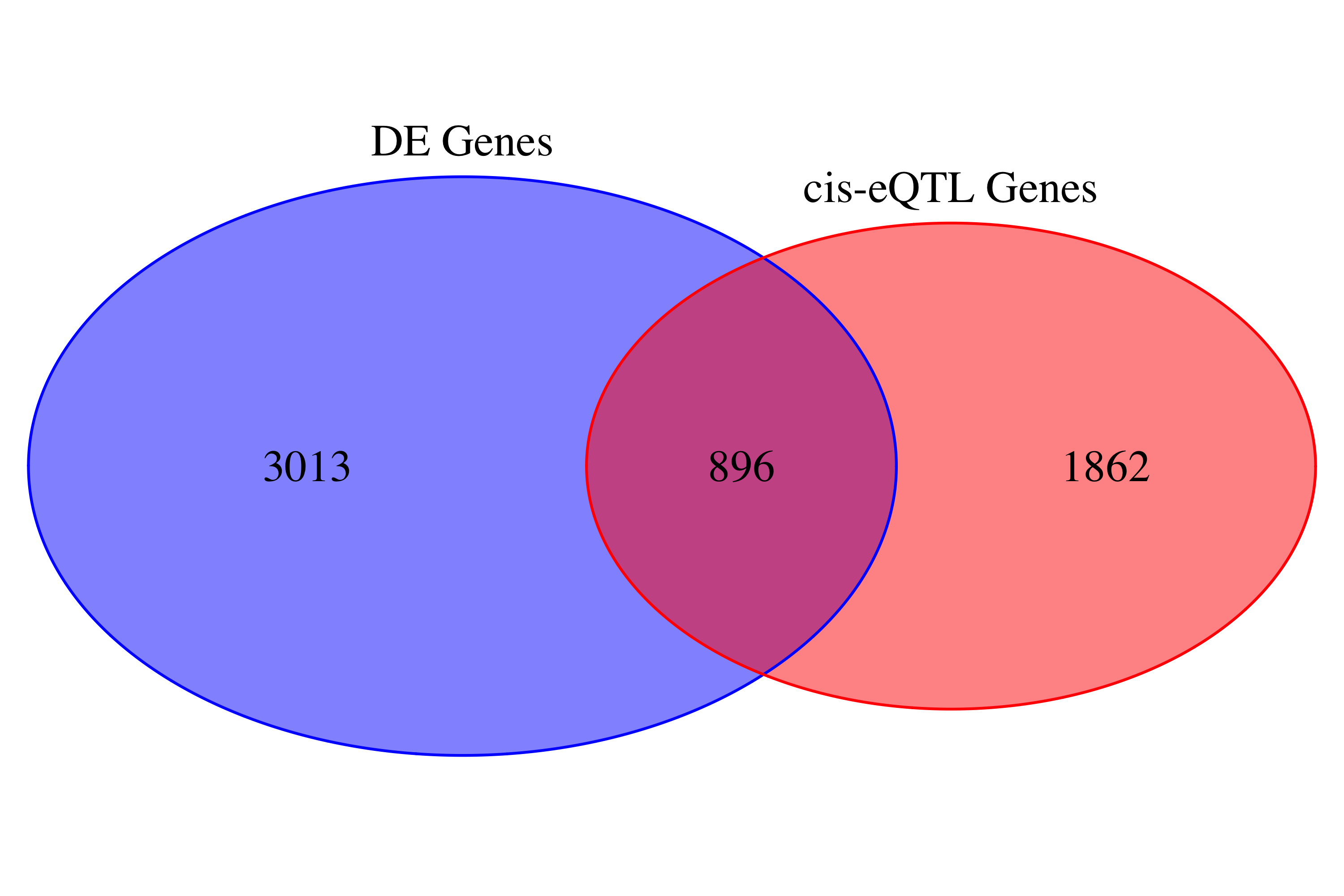


Figure S8. Venn diagram showing the overlap between unique genes identified as differentially expressed across stages of the menstrual cycle (blue) and unique genes with significant *cis*-eQTLs passing the FDR threshold of 0.05 (red).

**
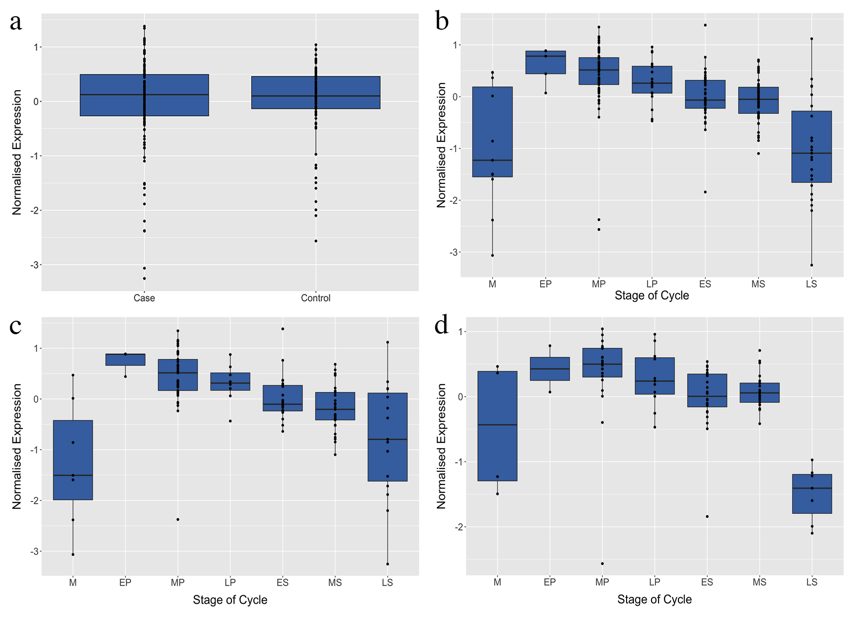
**

Figure S9. Expression of HOXA10 in endometrium. a) Expression in cases and controls. b) Expression across the seven stages of the menstrual cycle, menstruation (M), early-proliferative (EP), mid-proliferative (MP), late-proliferative (LP), early-secretary (ES), mid-secretary (MS) and late-secretary (LS). c) Expression in cases across the seven stages and d) expression in controls across the stages.

**
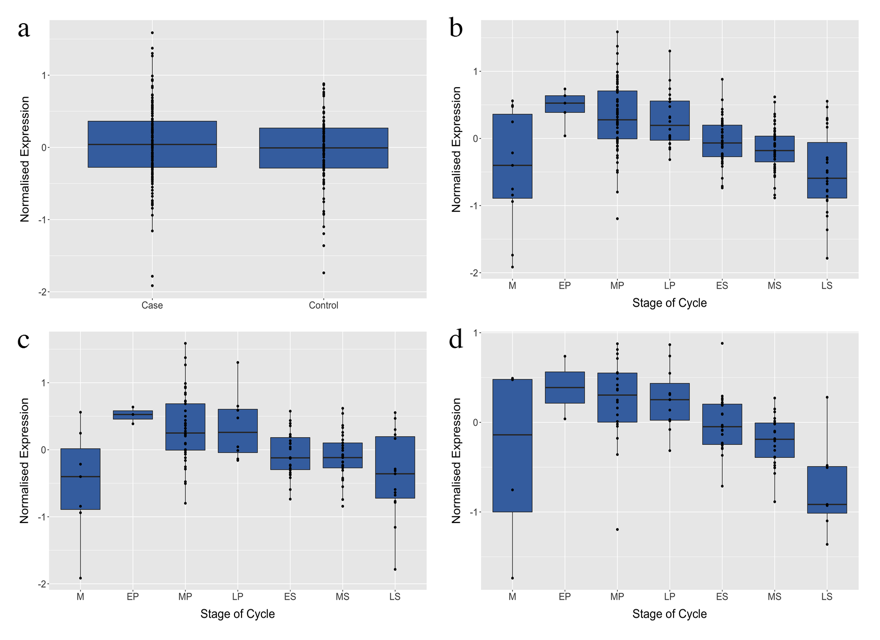
**

Figure S10. Expression of EMX2 in endometrium. a) Expression in cases and controls. b) Expression across the seven stages of the menstrual cycle, menstruation (M), early-proliferative (EP), mid-proliferative (MP), late-proliferative (LP), early-secretary (ES), mid-secretary (MS) and late-secretary (LS). c) Expression in cases across the seven stages and d) expression in controls across the stages.

**
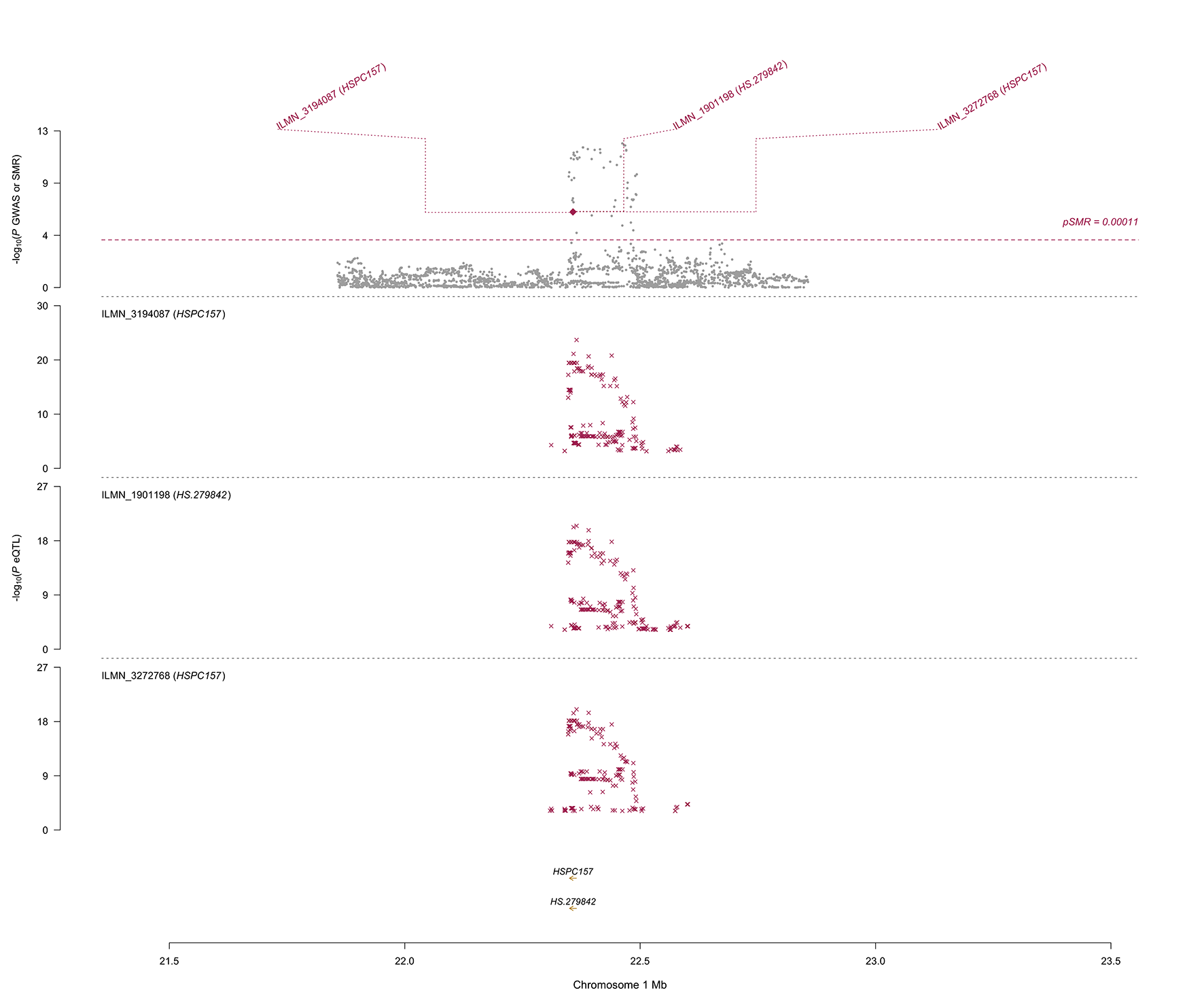
**

Figure S11. The SMR locus plot showing the results at the *HSPC157/HS.279842* (aka *LINC00339)* locus for Endometriosis. Top plot, grey dots represent the *P* values for SNPs from the latest GWAS meta-analysis for Endometriosis, diamond represents the *P* values for probes from the SMR test. Middle and bottom plots, the eQTL *P* values of SNPs from this study for the three probes tagging *LINC00339*.

**
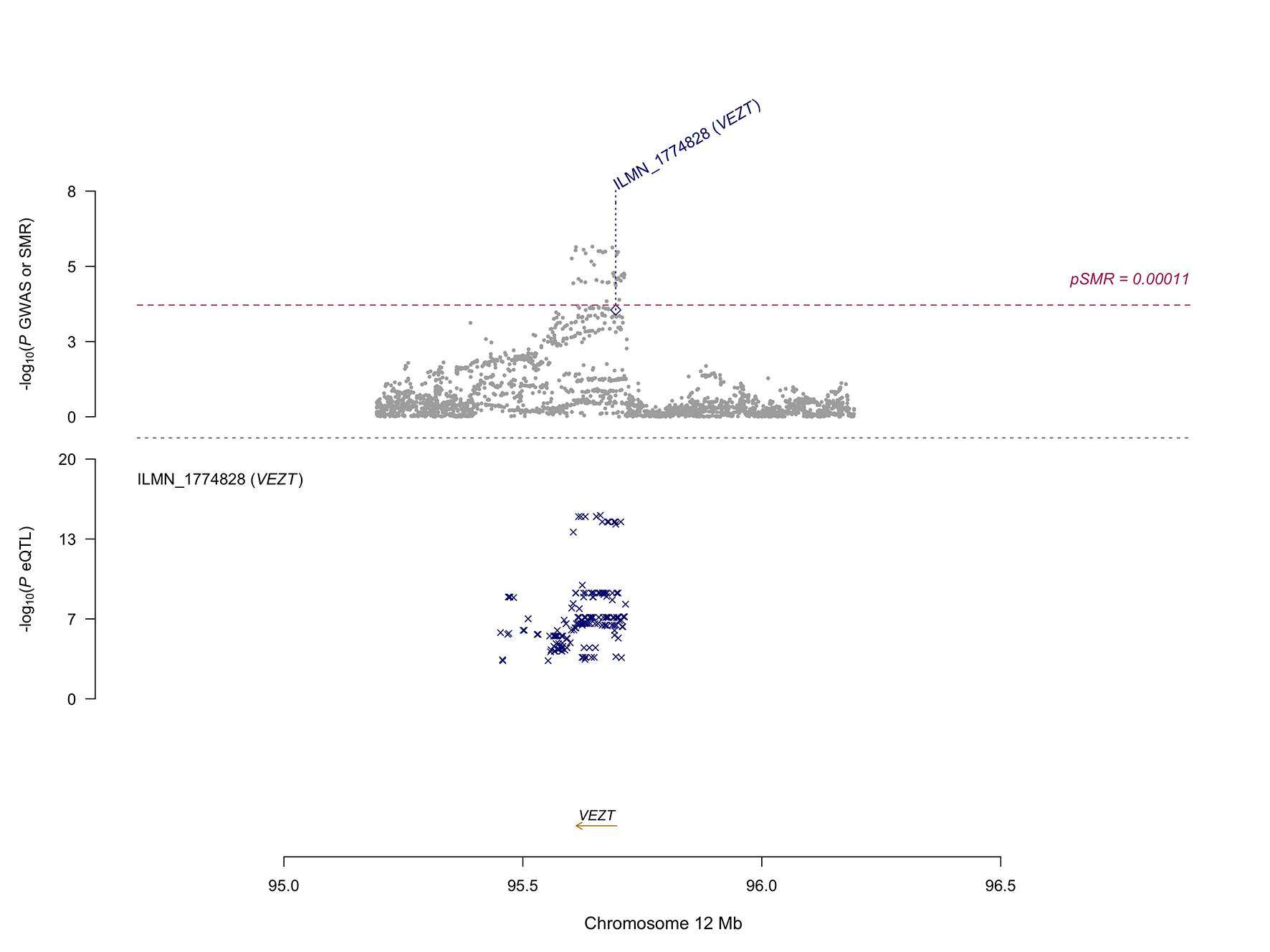
**

Figure S12. The SMR locus plot showing the results at the *VEZT* locus for Endometriosis. Top plot, grey dots represent the *P* values for SNPs from the latest GWAS meta-analysis for Endometriosis, diamond represents the *P* value for the probe from the SMR test. Bottom plot, the eQTL *P* values of SNPs from this study for the probe tagging *VEZT*.
